# Blockade of the calpain-1/HSP90/TGF-β1 axis by Astragaloside IV ameliorates bleomycin-induced pulmonary fibrosis

**DOI:** 10.3389/fphar.2026.1864914

**Published:** 2026-10-14

**Authors:** Silin Wei, Yu Liu, Bailin Tang, Yang Yu, Nan Sun, Meili Lu, Lili Sun, Guijun Wang

**Affiliations:** 1 Key Laboratory of Cardiovascular and Cerebrovascular Drug Research of Liaoning Province, Jinzhou Medical University, Jinzhou, China; 2 The First Affiliated Hospital of Jinzhou Medical University, Jinzhou, China; 3 The Third Affiliated Hospital of Jinzhou Medical University, Jinzhou, China

**Keywords:** Astragaloside IV, calpain-1, epithelial-mesenchymal transition, inflammation, oxidative stress, pulmonary fibrosis

## Abstract

**Background:**

Previous studies have confirmed the therapeutic potential of Astragaloside IV (AS-IV) in pulmonary fibrosis, yet the upstream regulatory mechanisms governing TGF-β1 stability, particularly involving proteolytic enzymes, are not fully understood. This study investigates the pivotal role of calpain-1 in modulating the HSP90/TGF-β1 signaling pathway, thereby elucidating the effects of AS-IV on bleomycin (BLM)-induced pulmonary fibrosis.

**Methods:**

Calpain-1 knockout (CAPN1^−/−^) and wild-type (WT) mice were used to establish pulmonary fibrosis models via intratracheal instillation of BLM. Both animal and cell models were administered varying doses of AS-IV for intervention. Inflammatory, oxidative stress, fibrotic, and epithelial-mesenchymal transition (EMT) markers were subsequently evaluated. Additionally, molecular docking, molecular dynamics simulations, and surface plasmon resonance (SPR) experiments were employed to assess the binding interactions between AS-IV and the calpain-1/HSP90/TGF-β1 axis.

**Results:**

The calpain-1/HSP90/TGF-β1 axis was significantly activated in BLM-induced pulmonary fibrosis mice. AS-IV ameliorated pulmonary fibrosis, EMT, oxidative stress, and inflammation while simultaneously suppressing this overactivated axis. Calpain-1 knockout inhibited HSP90 upregulation, attenuated TGF-β1/Smad2/3 activation, and reduced pulmonary fibrosis. *In vivo* experiments further confirmed the inhibitory effects of AS-IV and calpain-1 inhibition on lung epithelial cell injury and fibroblast proliferation. Notably, calpain-1 overexpression in MLE-12 cells abolished the protective effects of AS-IV against EMT, oxidative stress, and inflammation. Molecular docking, molecular dynamics simulations, and SPR experiments confirmed the direct binding between AS-IV and calpain-1, supporting its upstream regulatory role in this signaling axis.

**Conclusion:**

AS-IV attenuates pulmonary fibrosis by targeting calpain-1 to disrupt the HSP90/TGF-β1 signaling axis. These findings identify calpain-1 as a novel therapeutic target and support AS-IV as a promising candidate for PF treatment.

## 1 Introduction

Idiopathic pulmonary fibrosis (IPF) is a chronic, progressive interstitial lung disease characterized by excessive extracellular matrix accumulation, fibroblast activation, and irreversible lung scarring. The pathogenesis of IPF involves complex interactions among genetic, epigenetic, and environmental factors, with alveolar epithelial cell dysfunction playing a central role (Zoz et al., 2011; Liu et al., 2025). Epithelial-mesenchymal transition (EMT) is pivotal in the progression of pulmonary fibrosis, characterized by the invasion, migration, and extracellular matrix production by epithelial cells (Sadatpour et al., 2026). Concurrently, lung tissue in IPF patients exhibits elevated levels of reactive oxygen species (ROS), which inflict oxidative damage on epithelial cells, triggering inflammation and stimulating the release of proinflammatory cytokines. This cascade of events ultimately drives EMT and perpetuates fibrosis (Thannickal and Fanburg, 2000; Koli et al., 2008; Chatterjee and Chatterjee, 2020). Therefore, the discovery of safe and effective therapeutic agents targeting novel molecular pathways holds substantial clinical and translational significance for IPF intervention.

Astragaloside IV (AS-IV), a principal natural compound derived from the traditional Chinese herb Astragalus membranaceus, holds significant potential as a dietary or nutraceutical component for disease prevention1. Owing to its diverse pharmacological activities, AS-IV has garnered substantial attention for its anti-inflammatory, anti-fibrotic, antioxidant, and immunomodulatory effects (Xiao et al., 2026; Wang et al., 2024; Wu et al., 2026). Recent studies have highlighted its therapeutic potential in various fibrotic conditions, including PM2.5-induced lung injury via the NLRP3/caspase-1 axis, autophagy, and ferroptosis (Huang et al., 2022; Wang et al., 2022), as well as hypoxia-induced myocardial fibrosis through TRPM7 channel modulation (Lu et al., 2017). However, despite growing evidence supporting the antifibrotic efficacy of AS-IV in pulmonary fibrosis (Guan et al., 2024; Zhang et al., 2023; Zhu et al., 2024), the precise molecular mechanisms underlying its protective effects remain incompletely understood, particularly regarding upstream regulatory targets that govern TGF-β1 signaling stability.

Calpain-1, a Ca^2+^-dependent cysteine protease, catalyzes substrate-specific protein hydrolysis and plays crucial roles in regulating cell growth, differentiation, apoptosis, and cytoskeletal remodeling (Goll et al., 2003; Storr et al., 2011; Ono et al., 2016; Shapovalov et al., 2022; Averna et al., 2015). Notably, unlike calpain-2, calpain-1 specifically interacts with HSP90, a molecular chaperone known to stabilize TGF-β1 signaling components and promote collagen synthesis in fibrotic diseases (Averna et al., 2015; Li et al., 2016; Li et al., 2024). Emerging evidence has implicated calpain-1 in inflammatory regulation, EMT induction, and organ fibrosis through TGF-β1 pathway modulation (Tan et al., 2017; Jiang et al., 2012). In systemic sclerosis, HSP90 has been shown to stimulate TGF-β1 signaling, thereby driving myofibroblast differentiation (Tomcik et al., 2014). However, whether the calpain-1/HSP90/TGF-β1 signaling axis contributes to bleomycin(BLM)-induced pulmonary fibrosis, and whether this pathway serves as a direct therapeutic target for AS-IV, remains entirely unexplored.

In this study, using BLM-induced fibrotic models in both calpain-1 knockout and wild-type mice, combined with MLE-12 and MLF cell systems, we systematically elucidated the therapeutic mechanism of AS-IV. Through an integrated approach employing molecular biology techniques, molecular dynamics simulations, and surface plasmon resonance (SPR) technology, we demonstrate for the first time that AS-IV directly binds to calpain-1, thereby inhibiting HSP90-mediated TGF-β1/Smad2/3 activation and subsequent EMT, oxidative stress, and inflammation. Collectively, these findings not only reveal a novel mechanistic pathway underlying AS-IV’s antifibrotic effects but also identify calpain-1 as a previously unrecognized therapeutic target for pulmonary fibrosis, supporting AS-IV as a promising dietary or nutraceutical intervention for disease management.

## 2 Materials and methods

### 2.1 Reagents

Astragaloside IV was obtained from Nanjing Jingzhu Biotechnology Company (Z100023-1G, purity >98% as measured by HPLC, Nanjing, China). Mouse lung epithelial cells (MLE-12) and Mouse Lung Fibroblasts (MLF) were purchased from ATCC (American Type Culture Collection, Manassas, USA). Bleomycin (BLM), Cell Counting Kit-8, MDL-28170 (calpain-1 inhibitor), 17-AAG (HSP90 inhibitor) and SB525334 (TGF-β1 inhibitor) were obtained from Selleck Chemicals (Houston, TX, USA). Pirfenidone (PFD) was purchased from ApexBio (USA). Kits for measurement of TNF-α, IL-1β, IL-6, and CXCL1 were purchased from R&D System (USA). MDA, GSH, SOD, and CAT kits were purchased from Nanjing Jiancheng Technology Co., Ltd. The Annexin V-FITC Apoptosis Detection Kit was purchased from Solarbio (CA1020, Beijing, China). The Transwell 0.4 co-culture plate (Transwell-3379) was purchased from Corning Inc. (USA). Antibodies CAPN1 (10538-1-AP), HSP90 (13171-1-AP), E-cadherin (20874-1-AP), N-cadherin (22018-1-AP), Vimentin (10366-1-AP), collagen I (14695-1-AP), and Fibronectin (15613-1-AP) were from Proteintech (Wuhan, China). Antibodies TGF-β1 (A2124), Smad 2 (A11498), p-Smad 2 (AP0925), Smad 3 (A16913), p-Smad 3 (AP1263) and α-SMA (A17910) were obtained from ABclonal (Wuhan, China). Dihydroethidium (DHE) was purchased from Shanghai Yisheng Biotechnology Co., Ltd. (Shanghai, China).

### 2.2 Animal experiments

All animal experiments in this study were conducted in accordance with the guidelines of the Animal Experimentation Ethics Committee of Jinzhou Medical University (No. 2023031). The mice were housed in a controlled environment for 1 week (free access to food and water, 25 °C ± 2 °C). Seventy male wild-type C57BL/6 mice were randomly assigned to the following groups: normal group (Con), bleomycin group (BLM), AS-IV (L) group (BLM + Astragaloside IV low dose, 25 mg/kg/d), AS-IV (M) group (BLM + Astragaloside IV medium dose, 50 mg/kg/d), AS-IV (H) group (BLM + Astragaloside IV high dose, 100 mg/kg/d) (Yu et al., 2016) and PFD group (BLM + Pirfenidone, 300 mg/kg/d). Sixteen male calpain-1 knockout mice (Capn1^−/−^; C57BL/6N; Cyagen Biosciences, Inc.) were randomly assigned to two groups: the normal knockout group (CK: CAPN1 knockout) and the BLM tracheal drip knockout group (MK: BLM + CAPN1 knockout), with eight mice in each group. Mice were anesthetized with 1% pentobarbital sodium. After anesthesia, the mice were suspended to expose the trachea, and 5 mg/kg (50 μL) of BLM (dissolved in saline) was instilled into the trachea. Astragaloside IV was administered via oral gavage, with the compound dissolved in 0.5% sodium carboxymethylcellulose, at doses of 25, 50, and 100 mg/kg once daily for 3 weeks, starting on the first day following the tracheal instillation of BLM. After 3 weeks, lung tissues and bronchoalveolar lavage fluid from the mice were collected and stored at −80 °C for subsequent experiments.

### 2.3 Cell culture and transduction

MLE-12 mouse lung epithelial cells were sourced from the American Type Culture Collection (ATCC, Manassas, USA). The cells were cultured at 37 °C in a humidified incubator with 5% CO2. Dulbecco’s Modified Eagle Medium (DMEM, Gibco, USA), supplemented with 10% fetal bovine serum (Gibco, USA), was used for the culture. Following a 48-h incubation with bleomycin (BLM, 10 μg/mL), the cells were grouped as follows: control (Con), BLM (10 μg/mL), MDL-28170 (20 μM), 17-AAG (1 μM), SB525334 (1 μM), Pirfenidone (1.5 μM), and Astragaloside IV (40 μM) groups (Zhang et al., 2024; Liu et al., 2023). All groups were incubated for 48 h. To investigate the role of calpain-1 in the regulation of IPF, a CAPN1-lentiviral vector (pLV-CAPN1, GV367, Genechem Co., Ltd., Shanghai, China) was transduced into the MLE-12 cells for 12 h. The CAPN1-overexpression lentivirus had a functional titer of 2 × 10^7^ TU/mL. Following transduction, the cells were exposed to BLM for an additional 48 h. The success of calpain-1 overexpression was confirmed by Western blot analysis (Supplementary Figure S1).

### 2.4 Co-culture of MLE-12 cells with lung fibroblasts

MLE-12 cells and mouse lung fibroblasts (MLF cells) were harvested at a cell density of 80%–90%, digested with 0.25% trypsin (with EDTA) for 2 min, followed by the addition of 2 mL of complete medium to terminate the digestion. The cells were then centrifuged at 1,200 rpm for 3 min, and the supernatant was discarded. The cells were divided into three groups: the Con group, the BLM group, and the BLM + AS-IV group. A 0.4 μm, 24-well transwell co-culture plate was then used, with 0.5 mL of fibroblast suspension added to the upper layer and 1 mL of MLE-12 cell suspension added to the lower layer. The plate was incubated for 12 h.

### 2.5 Molecular docking, molecular dynamics simulations and surface plasmon resonance

Molecular docking analysis: small molecule-protein docking was performed using AutoDock (version 4.2.6)-Vina to investigate the relationship between Astragaloside IV and calpain-1, HSP90, and TGF-β1. Small molecule structures and protein structures were obtained from the PubChem (http://pubchem.ncbi.nlm.nih.gov, accessed 13 March 2024) and AlphaFold (http://alphafold.ebi.ac.uk, accessed 13 March 2024) databases, respectively. PyMOL (version 2.6) and AutoDock Vina were used to study the small molecule-protein interactions, as well as for further visualization and analysis.

In order to further investigate the complex interactions and their stability, molecular dynamics simulation (MD) technique was used in this study to simulate 100 ns protein-ligand complexes using GROMACS 2021.3 software (Da Silva et al., 2022). The AMBER99SB-ILDN force field was selected to create protein topology files. Small molecule ligand topology files were generated using Amber20 software and the GAFF force field (Man et al., 2017). A truncated n-octet TIP3P solvent cassette was added to the system at a distance of 10 nm, and Na/Cl was added to the system to equilibrate the system charge. Then, energy minimization was carried out using the 2500-step steepest descent method and the 2500-step conjugate gradient method (Ren et al., 2023). The NVT tethering simulation of 100 ps and the NPT equilibrium simulation of 100 ps were carried out at the system maintenance temperature of 298.15 K (Suleiman et al., 2021). Finally, the MD tethering simulation of 100 ns was carried out to calculate the long-range electrostatic interactions using the PME method, under the condition of periodic boundedness. The non-bonded truncation distance was set to 1 nm, the collision frequency was set to 2 ps, the system pressure was 101.325 kPa, the integration step was 2 s, and the trajectories were saved every 10 ps (Karabacak Atay et al., 2023). The free energy of binding between proteins and molecules was calculated by molecular dynamics simulation. Meanwhile, the last 20 ns of the equilibrium trajectory was extracted to stabilize the molecular conformation, which helps to understand the mode of interaction between proteins and molecules as well as the strength and type of interaction.

The activator is prepared by mixing 400 mM EDC and 100 mM NHS immediately before injection. The CM5 sensor chip is activated with the mixture at a flow rate of 10 μL/min for 420 s. CAPN1 is diluted to 20 μg/mL in immobilization buffer and then injected into the sample channel Fc2 at a flow rate of 10 μL/min, typically resulting in an immobilization level of 12,600 RU, while the reference channel Fc1 does not require a ligand immobilization step. The chip is deactivated by flowing 1 M ethanolamine hydrochloride at a flow rate of 10 μL/min for 420 s. Astragaloside IV is diluted to eight concentrations ranging from 7 to 500 μM in the same analysis buffer. Astragaloside IV is injected into the Fc1-Fc2 channel at a flow rate of 20 μL/min for a binding phase of 100 s, followed by a dissociation phase of 180 s. Both the binding and dissociation processes are carried out in the analysis buffer. The analysis is repeated for eight cycles in ascending order of analyte concentration. After each interaction analysis, the chip needs to be regenerated. SPR binding analysis was performed using a serial concentration series of Astragaloside A (7–500 μM). The KD value of 4.33 × 10^−4^ M was derived from fitting the sensorgrams of this representative experiment.

### 2.6 Histological and immunofluorescence

For histological examination, lung tissue was fixed in a 4% paraformaldehyde solution, followed by dehydration through paraffin embedding, sectioning, and a graded series of alcohol. The tissue sections were then analyzed for pathological alterations and collagen accumulation using hematoxylin and eosin (H&E) staining, Masson’s trichrome, and Sirius red staining. Observations were made under a Leica DMI 3000B light microscope to assess structural changes and fibrotic patterns within the lung tissue.

For immunofluorescence (IF) analysis, sections were deparaffinized, rehydrated, antigenically repaired, and then incubated with 0.5% Triton X-100 and blocked with 5% BSA for 1 h. Sections were incubated with anti-collagen I (1:100) primary antibody overnight at 4 °C. Nuclear counterstaining was performed using 4′,6′-diamidino-2-phenylindole (DAPI) for 5 min. The sections were subsequently imaged using a fluorescence microscope.

For immunofluorescence double staining, treated sections and cells were incubated with the primary antibody, mouse anti-SP-c (1:200), overnight at 4 °C, followed by incubation with the secondary antibody (red fluorescence) goat anti-mouse IgG (1:100) for 4 h. The sections were then blocked for 30 min and incubated with the primary antibody, rabbit anti-Vimentin (1:200), overnight at 4 °C. The following day, the sections were incubated with goat anti-rabbit IgG (green fluorescence) secondary antibody (1:100) for 4 h. Nuclei were counterstained with DAPI containing anti-fade solution for 5 min. The sections were then imaged using fluorescence microscopy.

### 2.7 Hydroxyproline detection

Mouse lung tissues were stored on ice for alkaline hydrolysis. The hydroxyproline concentration was measured using a hydroxyproline colorimetric assay kit, according to the manufacturer’s protocol (Nanjing Jiancheng Biological Co., Ltd., A030-2-1).

### 2.8 Measurement of reactive oxygen species

ROS levels were detected by dihydroethidium (DHE) staining. Briefly, fresh lung tissue sections and cultured MLE-12 cells were subjected to DHE staining strictly in accordance with the protocols provided by the reagent manufacturer. After incubation with DHE working solution in a light-protected environment, unbound probe was washed away. Fluorescence images were acquired under consistent parameter settings using a fluorescence microscope.

### 2.9 MDA, T-SOD, GSH, and SOD content measurement

The levels of MDA, T-SOD, and GSH in lung tissues and MLE-12 cells were determined using corresponding commercial biochemical assay kits. Briefly, tissue homogenates and cell lysates were prepared, and all detection procedures were carried out strictly following the manufacturer’s protocols. MDA reflects lipid peroxidation level, while T-SOD and GSH represent cellular antioxidant capacity. Finally, absorbance values were measured by a microplate reader to calculate the relative contents of each index among groups.

### 2.10 The collection of BALF and enzyme-linked immunosorbent assay (ELISA)

Anesthetized mice underwent bronchoalveolar lavage and were extubated using an indwelling needle. The lavage was performed three times with 0.5 mL of 4 °C phosphate-buffered saline (PBS). The supernatant was collected and stored at −80 °C after centrifugation at 1,500 rpm for 10 min. The levels of TNF-α, IL-1β, CXCL1, and IL-6 in the supernatants of mouse lung tissues, BALF, and MLE-12 cells were determined by ELISA using the respective ELISA kits according to the manufacturer’s protocol.

### 2.11 Cell viability and cytotoxicity assays

MLE-12 and MLF cells were seeded in 96-well plates at 1 × 10^5^ cells/well in 100 μL of culture medium and allowed to adhere. Cells were then treated with varying concentrations of MDL-28170, 17-AAG, SB525334, and Astragaloside IV, either alone or in combination, with vehicle-treated cells serving as controls. After 24 h of incubation, cell viability and cytotoxicity were evaluated using the Cell Counting Kit-8 (CCK-8) and the LDH Cytotoxicity Assay Kit (Beyotime Biotechnology, Shanghai, China) according to the manufacturer’s instructions. For the viability assay, 10 μL of CCK-8 reagent was added to each well, followed by a 2-h incubation at 37 °C. Absorbance was measured at 450 nm using a microplate reader, with cell-free wells serving as blanks. For the cytotoxicity assay, cell-free culture supernatants were collected to measure released LDH activity. Absorbance was read at 490 nm, and the percentage of cytotoxicity was calculated relative to the maximum LDH release in the control.

### 2.12 Cell proliferation assay

The proliferation of MLF cells derived from the MLE-12 and MLF co-culture system was evaluated using a commercial EdU proliferation detection kit (Beyotime, China). Briefly, MLF cells were separately collected from the co-culture model, resuspended in complete medium, and seeded evenly into 12-well plates for routine adherent culture. After corresponding experimental treatments, cell proliferation detection was performed strictly according to the manufacturer’s instructions. The EdU reagent can incorporate into newly synthesized DNA during cell proliferation, enabling specific fluorescent labeling of proliferative cells. Following standard incubation, staining, and washing steps, fluorescence images were acquired under identical microscopic parameters. The EdU-positive cell ratio was quantitatively analyzed to accurately evaluate the proliferative level of MLF cells in different groups.

### 2.13 Flow cytometry assay

Apoptosis was detected using the Annexin V-FITC Apoptosis Detection Kit according to the manufacturer’s instructions. MLF cells were plated in a 6-well plate. They were incubated with the indicated drugs for 24 h. The cells were collected, carefully washed three times with PBS, and then incubated with Annexin V-FITC staining reagent for 15 min in the dark.

### 2.14 Western blot analysis

Lung tissue, cultured MLE-12 cells, and MLF cells were all homogenized using a RIPA buffer solution. The supernatant was obtained by centrifuging the lysates at 12,000 rpm for 20 min at a temperature of 4 °C, followed by quantification of the protein content using a BCA Protein Assay Kit. Equal amounts of protein samples were loaded onto a 10% SDS-PAGE gel for electrophoresis, then transferred to a PVDF membrane, blocked with 5% skimmed milk for 120 min, and incubated overnight at 4 °C with the following primary antibodies: calpain-1 (1:10,000), HSP90 (1:10,000), TGF-β1 (1:5000), Smad2 (1:1,000), Smad3 (1:1,000), p-Smad2 (1:2000), p-Smad3 (1:1,000), VE-cadherin (1:2000), N-cadherin (1:1,000), Vimentin (1:10,000), α-SMA (1:1,000), collagen I (1:2000) and β-actin(1:10,000). After overnight incubation at 4 °C, the membranes were incubated with goat anti-rabbit IgG for 1.5 h at room temperature. The blot was visualized using a chemiluminescence system and the density of protein bands was quantified by ImageJ software.

### 2.15 Statistical analyses

All the data are presented as means ± SEM unless otherwise specified. For Western blot quantification, all data represent independent biological replicates, and sample sizes are modest. Key findings should be interpreted with appropriate caution. Reproducibility was supported by consistent results across independent experimental batches. The differences between two groups were assessed using a two-tailed unpaired Student’s t-test. Data were analyzed using one-way analysis of variance (ANOVA) followed by Tukey’s multiple comparisons test. Statistical analyses were performed using GraphPad Prism 9.0. Statistical significance was defined as a *p* value <0.05.

## 3 Results

### 3.1 Astragaloside IV and CAPN1 knockdown ameliorated BLM-induced lung fibrosis

To investigate whether Astragaloside IV exerts beneficial effects on IPF, we established a BLM-induced IPF model in wild-type and CAPN1 knockout mice. After 21 days of BLM exposure, the mice exhibited a significant decrease in body weight and a significant increase in lung weight (Figures 1E,F). Both the MK treatment group and the high-dose Astragaloside IV group significantly increased body weight and decreased lung weight. Lung tissues were stained with hematoxylin and eosin (H&E) to assess pathological alterations. The H&E staining results (Figure 1A) revealed significant thickening of the alveolar walls, loss of structural integrity, and a marked increase in inflammatory cells in the BLM group. Additionally, to assess collagen deposition in the lungs of mice treated with BLM, we performed Masson’s trichrome staining, Sirius red staining, and measured hydroxyproline (HYP) levels in lung tissue sections (Figures 1A–D). In the BLM-treated group, extensive collagen accumulation was observed, with a widespread distribution throughout the lung tissue. In comparison, the MK treatment group showed a marked reduction in histopathological alterations following BLM exposure. Additionally, Western blotting and IF results (Figures 1G–I) indicated that the MK group, as well as the Astragaloside IV treatment group, were effective in reducing the α-SMA and collagen I protein levels, markers of fibrosis.

**FIGURE 1:**
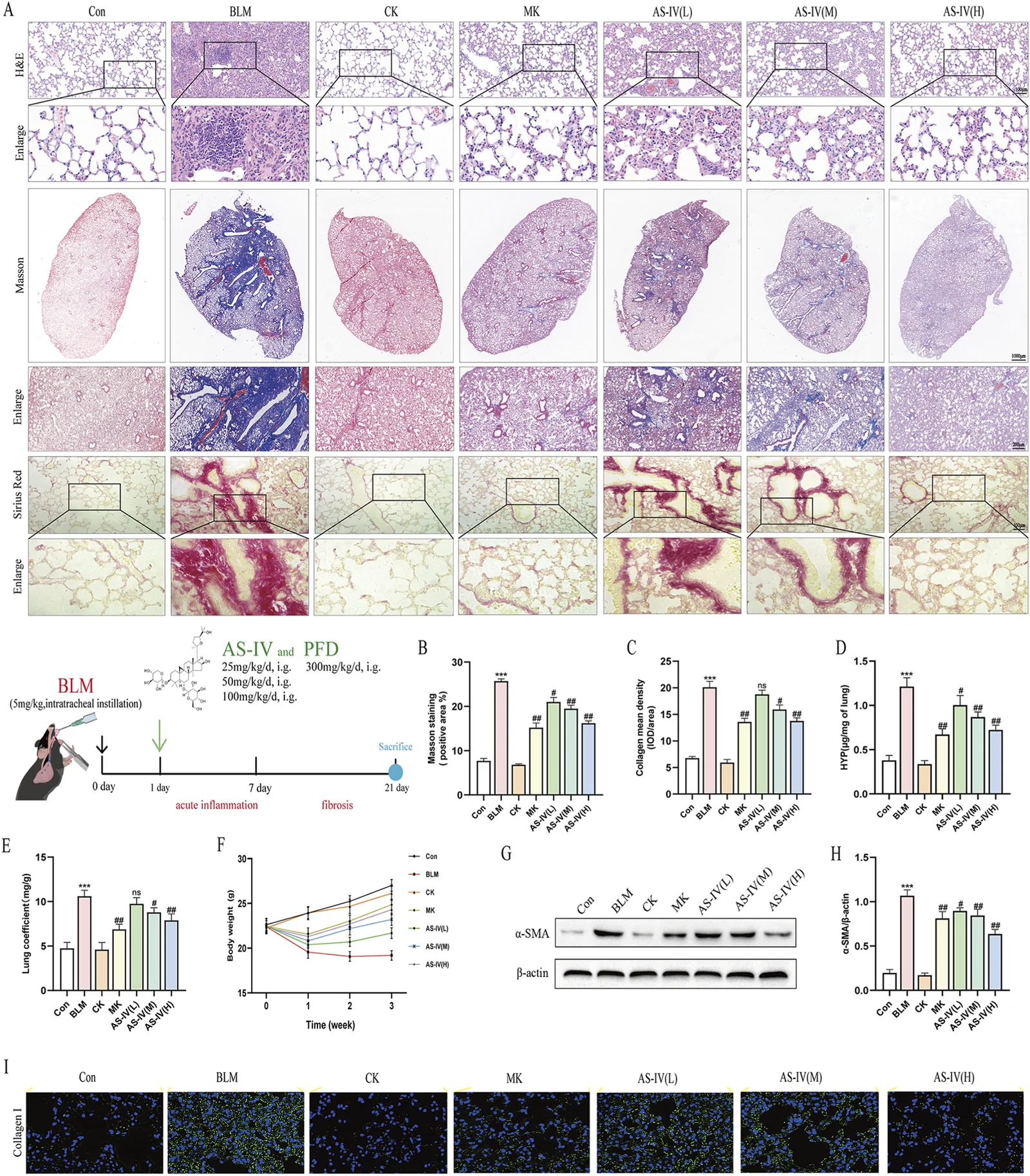
Astragaloside IV and CAPN1 knockdown ameliorated BLM-induced lung fibrosis. **(A)** The pathological changes in lung tissue were observed using H&E (top), Masson’s staining (middle), and Sirius red staining (bottom). Magnification: ×200, n = 3. **(B,C)** Masson’s staining and Sirius red staining quantitative analysis, n = 3. **(D)** The HYP content of lung tissue from mice in the various experimental groups, n = 8. **(E)** Lung coefficient (wet lung weight/body weight) of each group of mice, n = 8. **(F)** Changes in the weight of each group of mice over a 21-day period, n = 8. **(G,H)** Western blotting to detect α-SMA protein expression in lung tissues, n = 3. **(I)** Detection of collagen I expression by immunofluorescence. Magnification: ×200, n = 3. The data are expressed as the means ± SEM. ***P < 0.001 vs. the Con group, ^#^ *P* < 0.05, ^##^ *P* < 0.01 vs. the BLM group.

### 3.2 Astragaloside IV and CAPN1 knockdown ameliorated BLM-induced EMT

In the context of IPF, E-cadherin is crucial for maintaining lung cell integrity, while N-cadherin, vimentin, and fibronectin are associated with the pathological processes leading to lung tissue scarring and fibrosis. Compared to the Con group, Western blot analysis (Figures 2A–D) revealed a significant decrease in E-cadherin protein levels in BLM-induced IPF, while N-cadherin and vimentin protein levels significantly increased. However, the effects of these changes were significantly mitigated by CAPN1 knockdown and Astragaloside IV treatment. Immunohistochemistry (IHC) findings (Figure 2E) revealed a significant increase in fibronectin levels in the BLM-induced IPF group. This increase was effectively reduced by CAPN1 knockdown and Astragaloside IV treatment. Specifically, immunofluorescence (IF) double staining data indicated that the decrease in SP-C expression in the BLM group coincided with an increase in vimentin expression (Figure 2F; Supplementary Figure S2). CAPN1 knockdown and Astragaloside IV treatment were able to significantly reverse these outcomes. Additionally, the results (Supplementary Figures S3A-C) showed that both the high-dose Astragaloside IV group and the pirfenidone group significantly reduced the protein levels of N-cadherin and α-SMA, increased E-cadherin protein levels, and decreased EMT and fibrosis.

**FIGURE 2:**
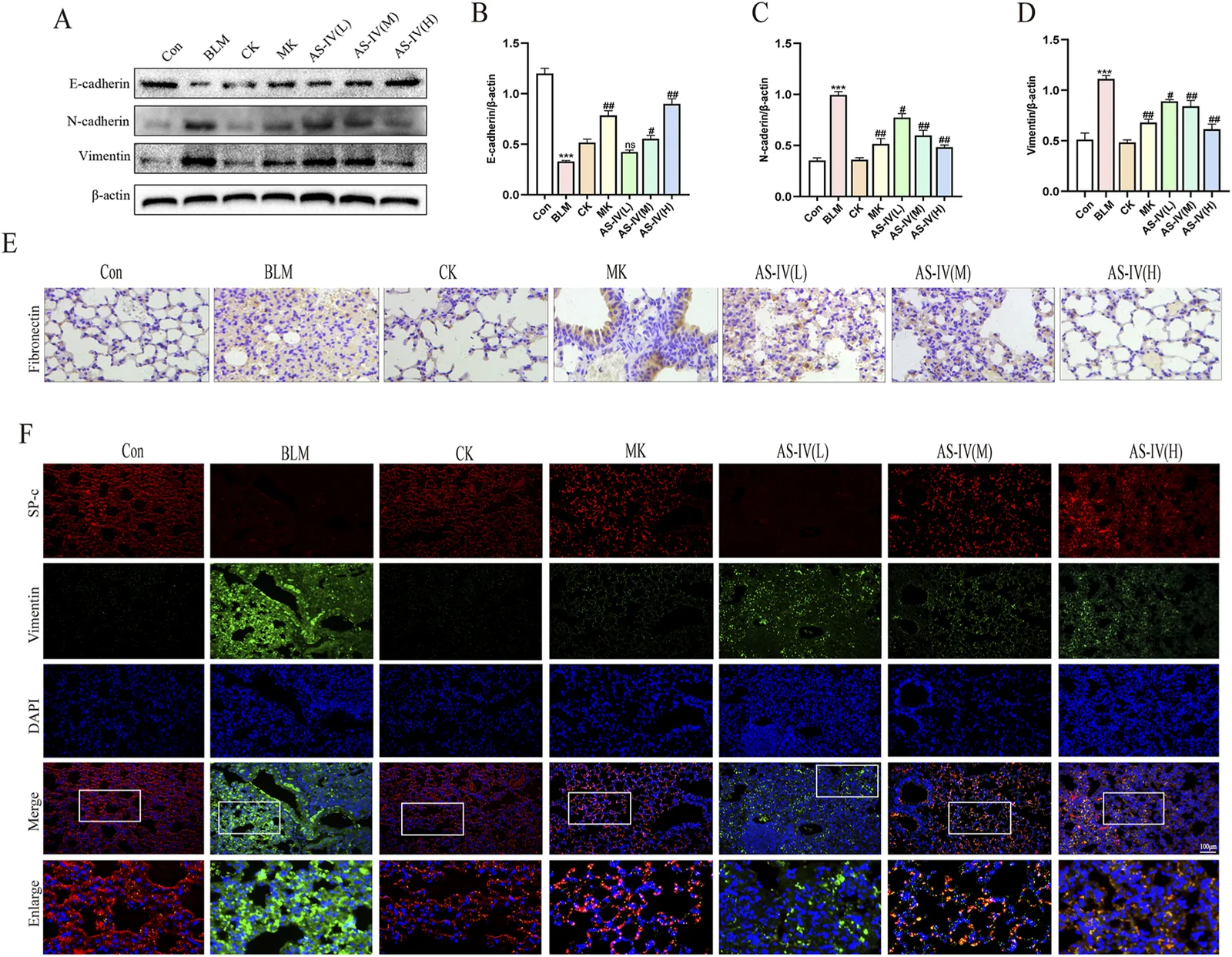
Astragaloside IV and CAPN1 knockdown ameliorated BLM-induced EMT.**(A–D)** Western blotting to detect E-cadherin, N-cadherin, and Vimentin protein expression in lung tissues., n = 3. **(E)** Detection of fibronectin proteins by immunohistochemistry. Magnification: ×200, n = 3. **(F)** Immunofluorescence double staining, SP-C (red), vimentin (green), and DAPI (blue). Magnification: ×200, n = 3. The data are expressed as the means ± SEM. ^***^ *P* < 0.001 vs. the Con group, ^#^ *P* < 0.05, ^##^ *P* < 0.01 vs. the BLM group.

### 3.3 Astragaloside IV and CAPN1 knockdown dampened oxidative stress and inflammation in BLM-induced IPF

To investigate the effects of Astragaloside IV and CAPN1 on oxidative stress and inflammation induced by BLM, we performed DHE staining and assays to measure ROS, MDA, SOD, GSH, and CAT. Additionally, ELISA kits were used to measure the levels of inflammatory mediators in both BALF and lung tissue samples. In mice treated with BLM (Figure 3A), a substantial increase in ROS within lung tissue was observed. This increase was significantly reduced by both Astragaloside IV treatment and CAPN1 knockdown. The results from the assays showed that in the BLM group (Figures 3B–E), MDA levels were increased in the lung tissues of the mice, whereas SOD, GSH, and CAT levels were reduced. However, Astragaloside IV treatment and CAPN1 knockdown exhibited a marked decrease in lipid oxidation and an increase in antioxidant activity in the lung tissues of the mice. The expression levels of TNF-α, IL-1β, IL-6, and CXCL1 in BALF and lung tissue were significantly increased in the BLM group (Figures 3F–M). However, the alterations in the Astragaloside IV and CAPN1 knockdown treatment groups were considerably diminished. Furthermore, to explore the therapeutic effect of Astragaloside IV on IPF, pirfenidone was used as a comparison. The results showed that both the high-dose Astragaloside IV group and the pirfenidone group significantly inhibited inflammation (Supplementary Figures S3B-G) and, simultaneously, reduced MDA levels and increased T-SOD and GSH levels, which were also significant.

**FIGURE 3:**
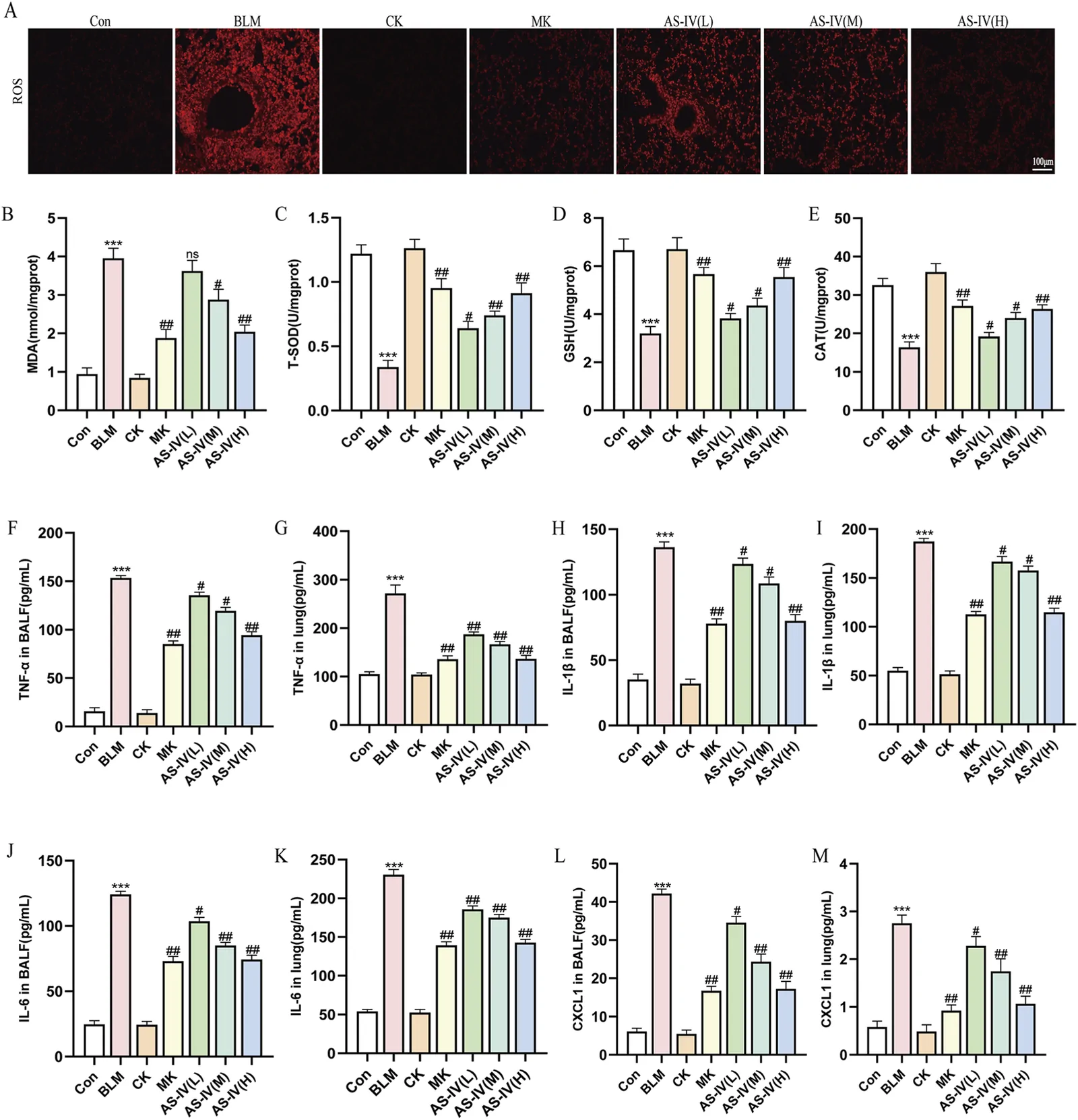
Astragaloside IV and CAPN1 knockdown could dampen oxidative stress and inflammation in BLM-induced IPF. **(A)** DHE staining was used to detect intercellular ROS levels in lung tissue. Magnification: ×200, n = 3. **(B)** MDA, **(C)** T-SOD, **(D)** GSH, and **(E)** CAT kits for detection of oxidative stress levels in lung tissue homogenates, n = 8. **(F–M)** ELISA kits were used to check TNF-α, IL-1β, IL-6, and CXCL1 levels in mouse BALF and lung tissue homogenates, n = 8. The data are expressed as the means ± SEM. ***P < 0.001 vs. the Con group, ^#^ *P* < 0.05, ^##^ *P* < 0.01 vs. the BLM group.

### 3.4 Astragaloside IV inhibited CAPN1 expression and inhibited the activation of the HSP90/TGF-β1 signaling pathway in BLM-induced IPF

Results from Western blot analysis (Figures 4A–G) showed significant increase in calpain-1, HSP90, TGF-β1, p-Smad2, and p-Smad3 protein levels in BLM-induced IPF, while treatment with Astragaloside IV and CAPN1 knockdown reduced the levels of calpain-1, HSP90, TGF-β1, p-Smad2, and p-Smad3 proteins. Also, Western blot analysis confirmed that CAPN1 protein expression was markedly reduced in lung tissues from CAPN1-KO mice, verifying the effective depletion of CAPN1 at the protein level in the animal model used in this study (Figures 4A,B). To further explore the mechanism of action of Astragaloside IV in BLM-induced pulmonary fibrosis, we performed molecular docking studies. The molecular docking results (Figure 4H) showed that Astragaloside IV stably interacted with calpain-1, HSP90, and TGF-β1 proteins, with docking energies of (−8.26 to −7.6), (−7.8 to −7.4), and (−9.6 to −8.7) kcal/mol, respectively. These binding energies are all less than −7 kcal/mol, demonstrating that the binding of the three protein-Astragaloside IV complexes is relatively stable.

**FIGURE 4:**
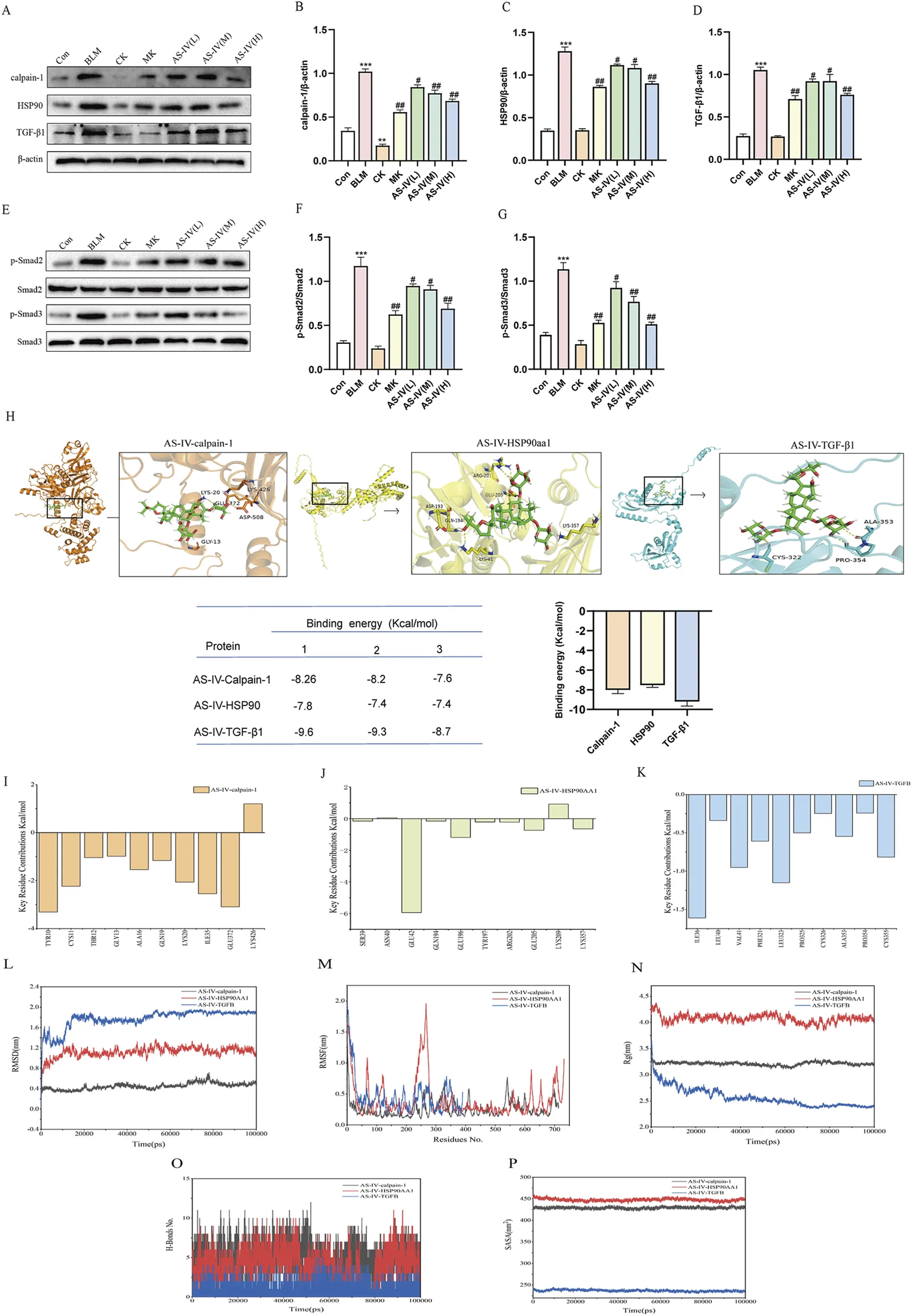
Astragaloside IV reduced CAPN1 expression and inhibited the activation of HSP90/TGF-β1 signaling pathway in BLM-induced IPF. **(A–G)** Western blotting was utilized to explore the protein expression levels of calpain-1, HSP90, TGF-β1, p-Smad2, p-Smad3, Smad2, and Smad3, n = 3. **(H)** Molecular docking. The left figure shows the overall view, and the right figure shows the partial view. Astragaloside IV (green), calpain-1 (orange), HSP90 (yellow), TGF-β1 (blue), and yellow dashed lines are hydrogen bonds. Molecular dynamics simulations. **(I–K)** MMPBSA analysis of the decomposition energies of key hydrogen bonded residues. **(L)** Atomic root mean square deviation (RMSD) of the complexes. **(M)** Root mean square fluctuation (RMSF) of the complexes. **(N)** Radius of gyration (Rg) of the complexes. **(O)** Changes in the number of hydrogen bonds (H-bonds) in the complexes. **(P)** Volumizable surface area (SASA) of the complexes.

To thoroughly examine the interaction between the receptor proteins and Astragaloside IV, and to verify the dynamic stability of the system, we performed 100 ns molecular dynamics simulations of the complex. MM-PBSA (Molecular Mechanics/Poisson-Boltzmann Surface Area) results (Figures 4I–K) showed that AS-IV-calpain-1 (ΔG = −69.91 kJ/mol) > AS-IV-TGF-β1 (ΔG = −63.50 kJ/mol) > AS-IV-HSP90 (ΔG = −28.21 kJ/mol), implying that the binding energy of the ligand to the target receptor increased across different active sites, thereby enhancing the stability of the complex. Root Mean Square Deviation (RMSD) results (Figure 4L) indicated that after approximately 15 ns of molecular dynamics simulation, the systems began to stabilize, with overall fluctuations remaining relatively smooth and free from any significant large-scale motion or structural alterations. Root Mean Square Fluctuation (RMSF) results (Figure 4M) showed that no amino acid residues exceeded an RMSF value of 2.0 nm. During the simulations, the stability of all amino acids was found to be excellent. In particular, the RMSF values in the hydrophobic and stable areas were significantly lower than in other regions, indicating that the AS-IV-calpain-1 system exhibits enhanced structural stability in these areas. The results (Figure 4N) showed that as the simulation time progressed, the Rg value of each system gradually decreased, suggesting a more compact protein structure and an increasingly stable molecular weight. Hydrogen bonding is widely recognized as crucial for stabilization. The results (Figure 4O) showed that the number of hydrogen bonds in the three systems remained relatively stable throughout the 100 ns simulation. The results (Figure 4P) showed that the buried surface area of Astragaloside IV with calpain-1, HSP90, and TGF-β1 systems was stabilized at 21–430 nm^2^, 448–452 nm^2^, and 235–240 nm^2^, respectively, suggesting that hydrophobic residues play an important role in protein folding and contribute to forming a hydrophobic core, which helps maintain the stability of proteins. Consequently, it can be inferred that Astragaloside IV can stably bind to calpain-1, HSP90, and TGF-β1.

### 3.5 Astragaloside IV ameliorated cell damage and EMT in MLE-12 cells through inhibiting calpain-1/HSP90/TGF-β1 signaling pathway

To confirm the therapeutic efficacy of Astragaloside IV for pulmonary fibrosis, MDL-28170, 17-AAG, SB525334, and Astragaloside IV were used in in vitro experiments. The CCK-8 assay results (Figure 5A) indicated that a 40 μM concentration of Astragaloside IV notably improved the viability of MLE-12 cells. Consequently, this concentration was chosen for use in the following experiments. The results from JC-1 staining (Figures 5B,C) demonstrated that Astragaloside IV effectively enhanced the mitochondrial membrane potential in MLE-12 cells compromised by BLM exposure, thereby mitigating cellular damage. Furthermore, LDH assay results (Figure 5D) indicated that Astragaloside IV, along with MDL-28170, 17-AAG, and SB525334, were effective in mitigating damage to MLE-12 cells caused by BLM. Western blot analysis (Figures 5D–I) revealed that Astragaloside IV markedly enhanced E-cadherin protein levels while suppressing the levels of N-cadherin, vimentin, α-SMA, and collagen I proteins. The therapeutic impact of Astragaloside IV was similar to that of MDL-28170, 17-AAG, and SB525334. Notably, IF double staining (Figure 5J) indicated that Astragaloside IV upregulated the expression of SP-C and downregulated vimentin expression. The results shown in (Supplementary Figure S4A) indicate that both the Astragaloside IV and pirfenidone groups significantly improved the expression of EMT and fibrosis proteins.

**FIGURE 5:**
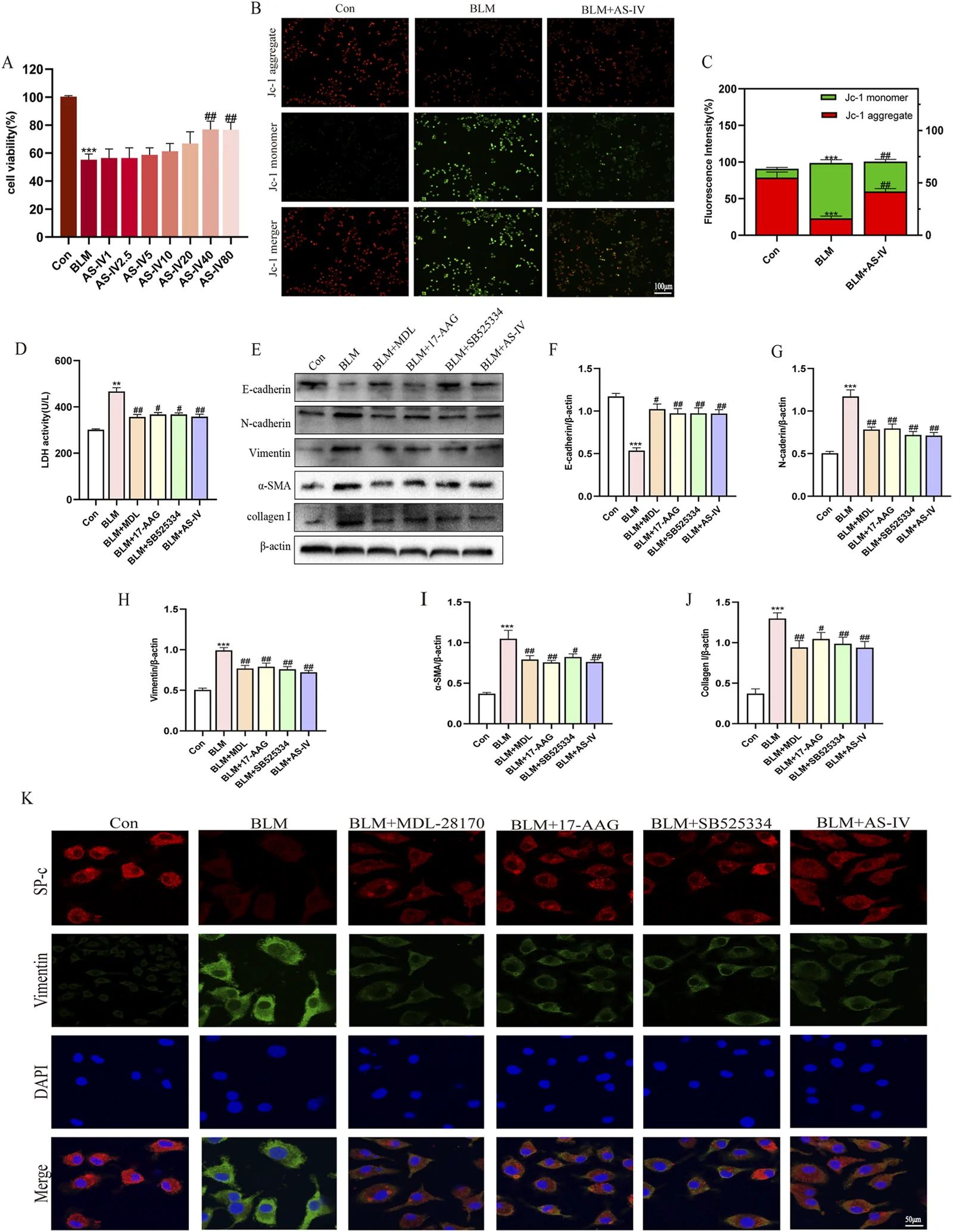
Astragaloside IV ameliorates BLM-induced fibrosis and EMT in MLE-12 cells through inhibiting calpain-1/HSP90/TGF-β1 signaling pathway. **(A)** CCK8 assay to detect the effect of Astragaloside IV on the viability of MLE-12 cells, n = 8. **(B,C)** JC-1 staining was used to detect damage to MLE-12 cells. Magnification: ×200, n = 3. **(D)** The LDH kit measures cellular damage in different group, n = 8. **(E–J)** Western blotting of MLE-12 cells was used to measure the protein expression of E-cadherin, N-cadherin, Vimentin, α-SMA and collagen I, n = 3. **(K)** Immunofluorescence detection of SP-c and Vimentin proteins in MLE-12 cells. Magnification: ×200, n = 3. The data are expressed as the means ± SEM. ^*^ *P* < 0.05, ^**^ *P* < 0.01,^***^ *P* < 0.001 vs. the Con group, ^#^ *P* < 0.05, ^##^ *P* < 0.01 vs. the BLM group.

### 3.6 Astragaloside IV reduced oxidative stress and inflammation in lung epithelial cells by BLM by blocking the calpain-1/HSP90/TGF-β1 pathway

To investigate the reduction of BLM-induced oxidative stress and inflammation in MLE-12 cells by inhibiting the calpain-1/HSP90/TGF-β1 pathway, we used DHE, MDA, SOD, GSH, CAT, and ELISA kits. The findings (Figures 6A–H) indicated that Astragaloside IV increased the concentrations of SOD, GSH, and CAT while decreasing the concentrations of ROS, MDA, TNF-α, IL-6, and IL-1β. These outcomes were comparable to those observed with MDL-28170, 17-AAG, and SB525334. Furthermore, the results from Supplementary Figures S4B-G indicate that both the Astragaloside IV and pirfenidone groups significantly inhibited the levels of MDA, TNF-α, IL-1β, and IL-6, and increased the levels of T-SOD and GSH.

**FIGURE 6:**
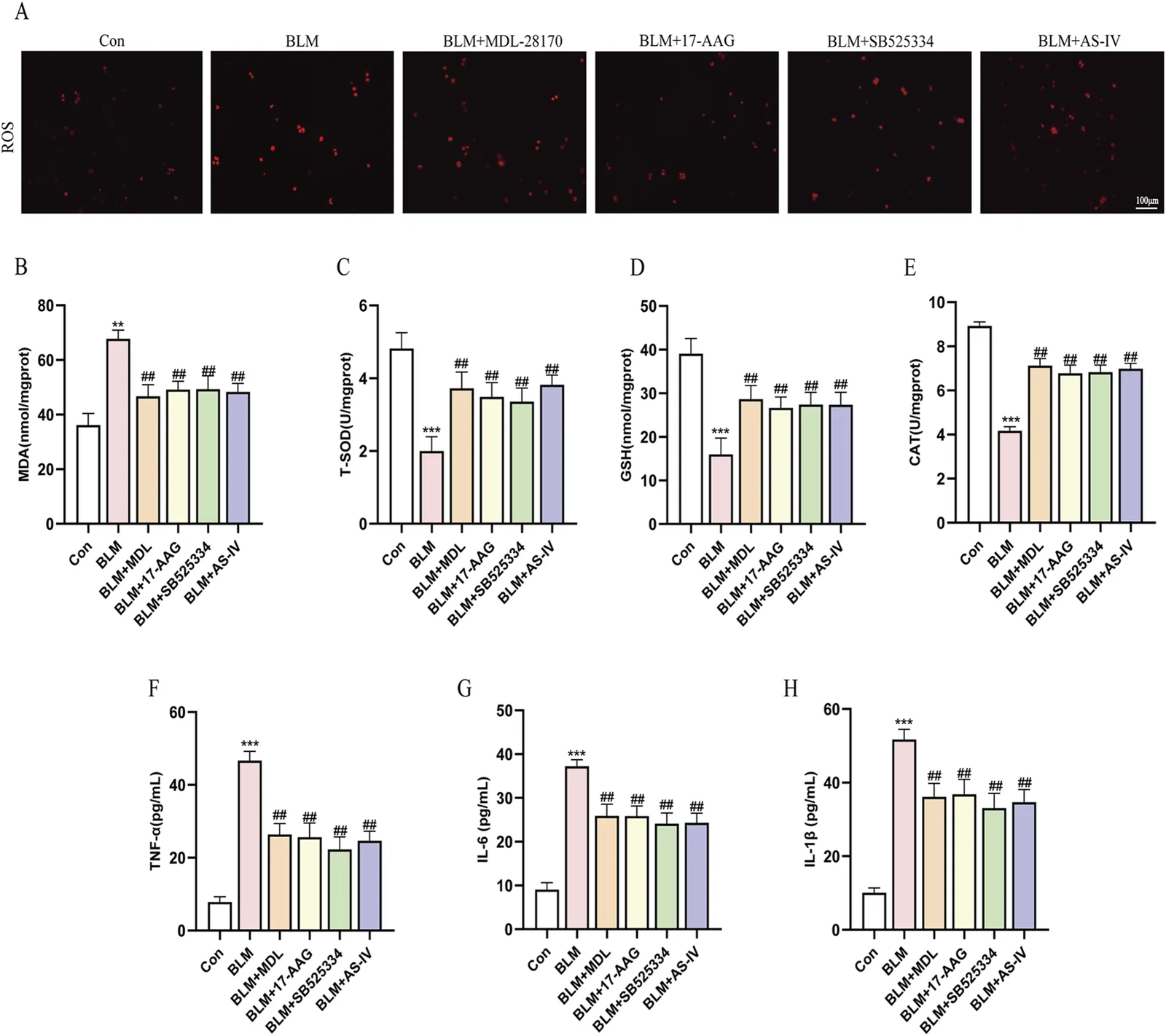
Astragaloside IV reduced oxidative stress and inflammation in lung cells damaged by BLM by blocking the calpain-1/HSP90/TGF-β1 pathway. **(A)** DHE detection of ROS levels in MLE-12 cells. Magnification: ×200, n = 3. **(B–E)** MDA, T-SOD, GSH, and CAT expression levels were detected in MLE-12 cells using the kits, n = 8. **(F–H)** Levels of TNF-α, IL-1β, and IL-6 in MLE-12 cell culture supernatants were assayed with ELISA kits, n = 8. The data are expressed as the means ± SEM. ^**^ *P* < 0.01, ^***^ *P* < 0.001 vs. the Con group, ^##^ *P* < 0.01 vs. the BLM group.

### 3.7 Astragaloside IV inhibited activation of calpain-1/HSP90/TGF-β1 signaling pathway in BLM-induced lung epithelial cells

To confirm whether Astragaloside IV affects the calpain-1/HSP90/TGF-β1 pathway in BLM-exposed MLE-12 cells, Western blot analysis was performed. The results (Figures 7A–G) demonstrated that Astragaloside IV decreased the protein levels of calpain-1, HSP90, TGF-β1, and p-Smad2 and p-Smad3. Additionally, the effects of Astragaloside IV were similar to those observed in the MDL-28170, 17-AAG, and SB525334 treatment groups. The results of the surface plasmon resonance experiment showed (Figure 7G) that calpain-1 binds to Astragaloside IV, with a dissociation equilibrium constant of 4.33 × 10^−4^ M (Figure 7H).

**FIGURE 7:**
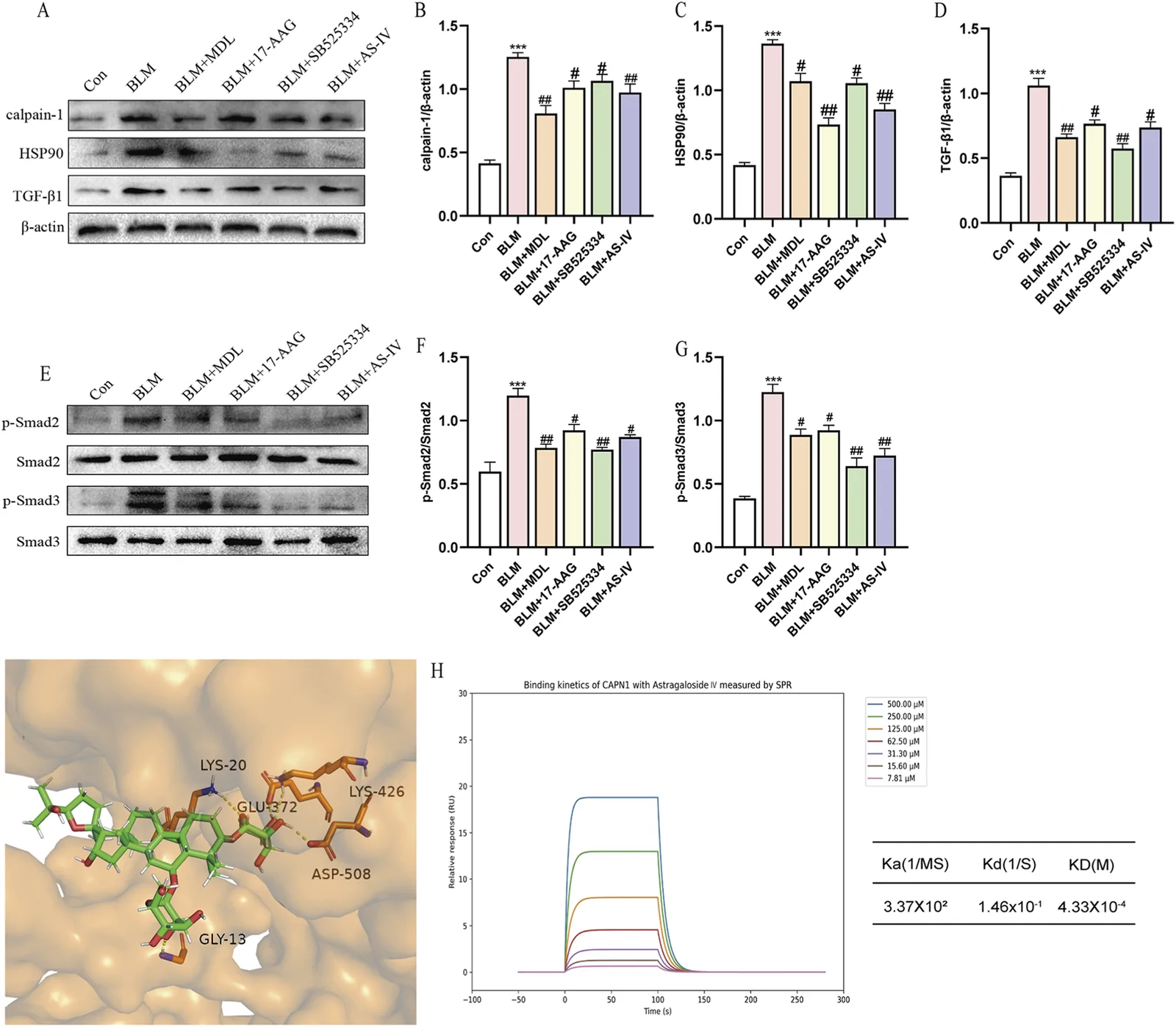
Astragaloside IV inhibited activation of calpain-1/HSP90/TGF-β1 signaling pathway in BLM-induced lung epithelial cells. **(A–G)** Western blotting technique was employed to test calpain-1, HSP90, TGF-β1, p-Smad2, p-Smad3, Smad2, and Smad3 protein levels in different groups of MLE-12 cells, n = 3. **(H)** SPR analysis of Astragaloside IV binding to calpain-1. Recombinant human calpain-1 protein was immobilized on an activated sensor chip, followed by flowing a series of concentrations of Astragaloside IV over the chip. The data are expressed as the means ± SEM. ^***^ *P* < 0.001 vs. the Con group, ^#^ *P* < 0.05, ^##^ *P* < 0.01 vs. the BLM group.

### 3.8 Astragaloside IV improved BLM-induced proliferation of MLF cells

To elucidate the mechanism by which Astragaloside IV exerts its effects and to mitigate the impact of MLE-12 cells on the proliferation of MLF cells, we established a co-culture system involving both MLE-12 and MLF cells (Figures 8A). EdU staining and flow cytometry analyses (Figures 8B,C) indicated that in the BLM group, there was a notable increase in MLF cell proliferation and a concurrent reduction in apoptosis. Importantly, the administration of Astragaloside IV effectively reversed these effects. Western blot analysis (Figures 8D,E) revealed a significant increase in the levels of fibrosis markers, α-SMA and collagen I, in MLF cells. However, in the Astragaloside IV treatment group, the fibrotic response in MLF cells was reduced.

**FIGURE 8:**
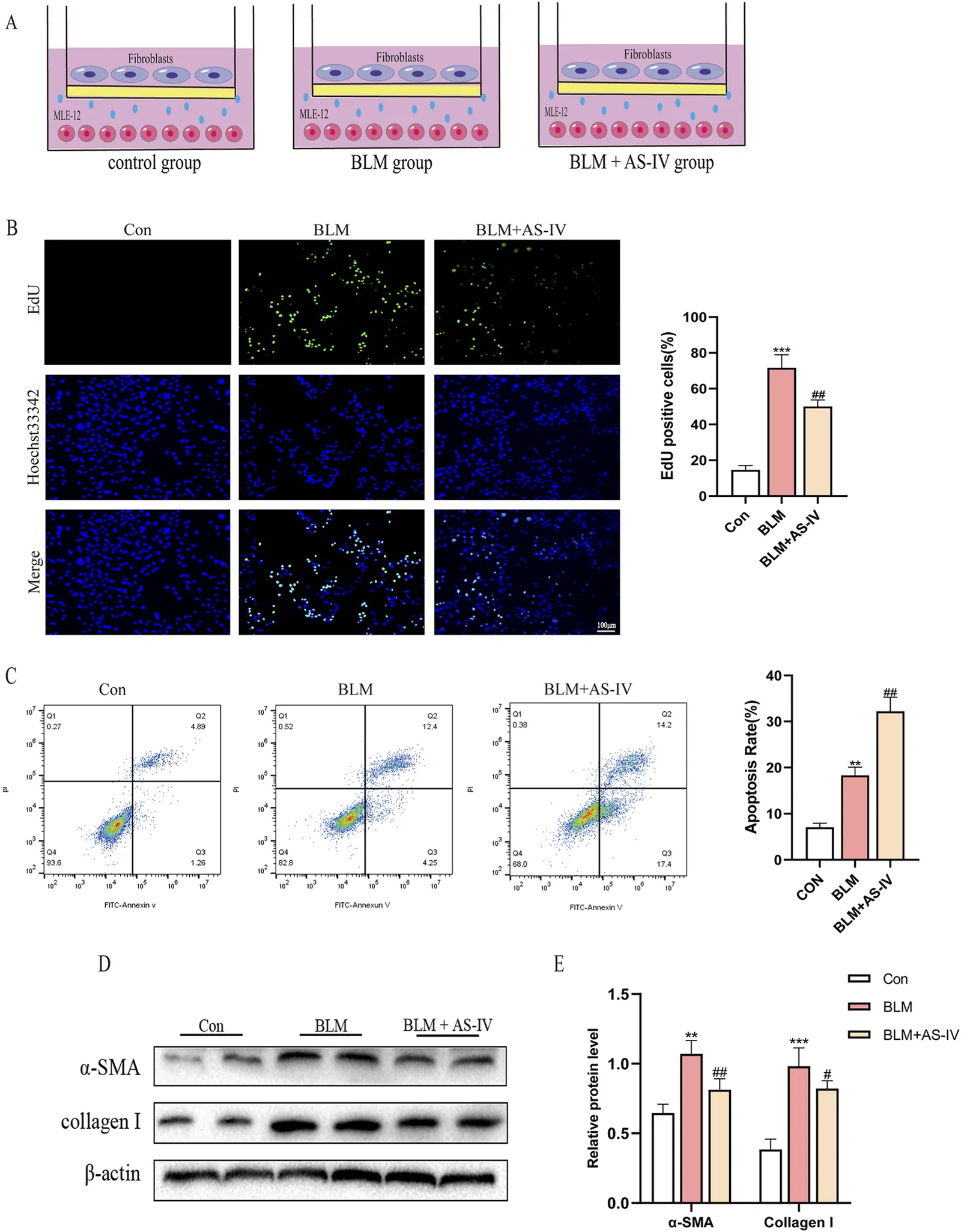
Astragaloside IV improved BLM-induced proliferation of MLF cells and MLE-12 cell damage. **(A)** CCK8 assay for cell viability at different AS-IV concentrations, n = 8. **(B)** Edu kit assay for MLF cell proliferation. Magnification: ×200, n = 3. **(C)** Flow cytometry was used to detect apoptosis in MLF cells, n = 3. **(D,E)** Western blotting was used to detect the expression of α-SMA and collagen I proteins in MLF cells (n = 3). The data are expressed as the means ± SEM. ^*^ *P* < 0.05, ^**^ *P* < 0.01,^***^ *P* < 0.001 vs. the Con group, ^#^ *P* < 0.05, ^##^ *P* < 0.01 vs. the BLM group.

### 3.9 Overexpression of calpain-1 reversed the effects of astragaloside IV on oxidative stress, inflammation, and EMT in BLM-induced fibrosis

To further elucidate the mechanism of action of Astragaloside IV in BLM-induced IPF, lentiviral infection was employed to overexpress CAPN1 in MLE-12 cells. When CAPN1 was overexpressed, the effects of Astragaloside IV on MLE-12 cells were reversed. This included its ability to reduce cell damage and increase the levels of SOD, GSH, and CAT, as well as its capacity to lower MDA, ROS, TNF-α, IL-1β, and IL-6 levels (Figures 9A–I). Additionally, the results of the Western blot analysis (Figures 9J–T) showed that the ability of Astragaloside IV to increase E-cadherin and decrease N-cadherin, vimentin, α-SMA, calpain-1, HSP90, TGF-β1, p-Smad2, and p-Smad3 was abolished by overexpression of calpain-1.

**FIGURE 9:**
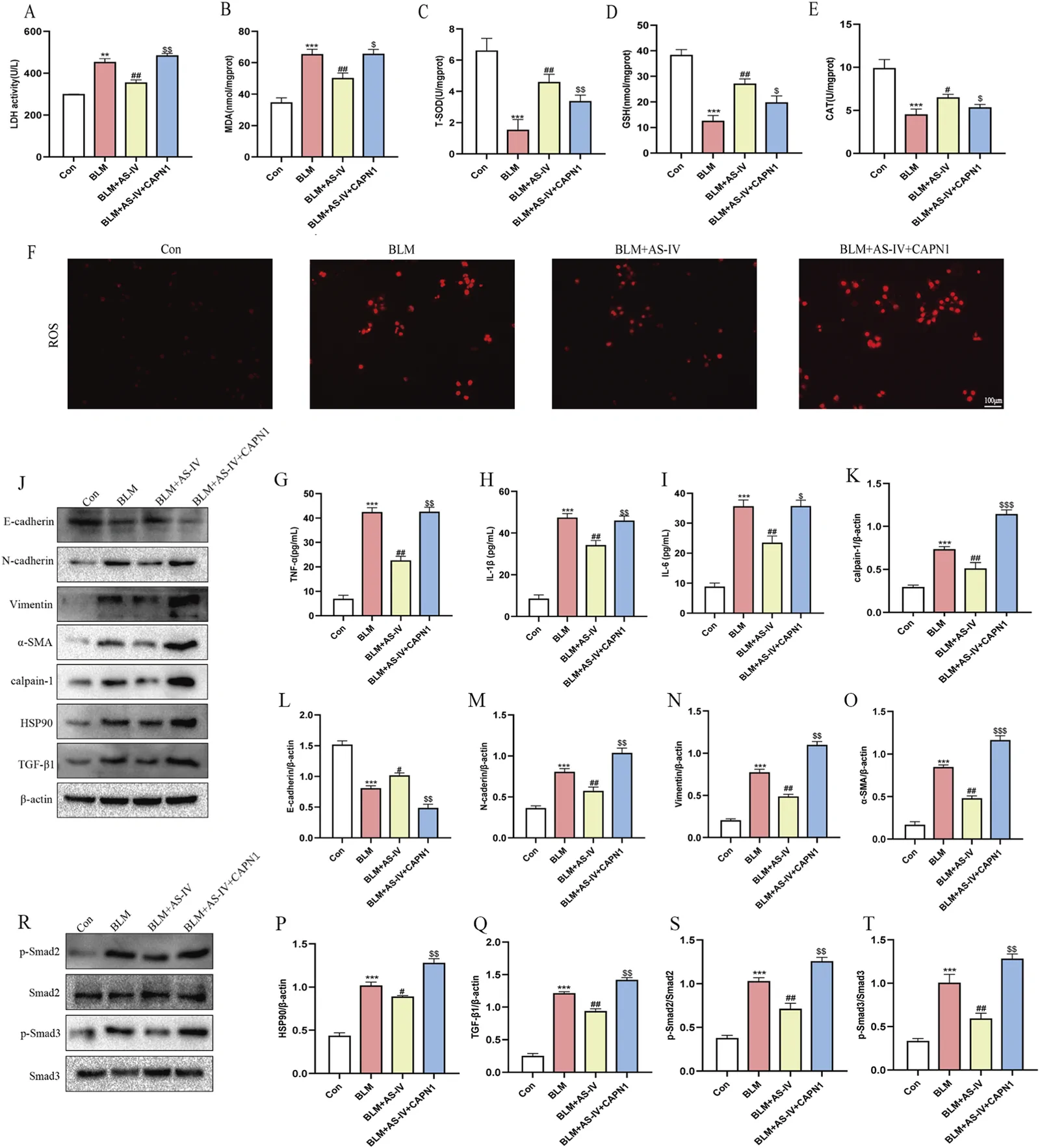
Overexpression of calpain-1 reverses the effects of Astragaloside IV on oxidative stress, inflammation, and EMT in BLM-induced fibrosis. **(A)** LDH assay for MLE-12 cell damage in different subgroups, n = 8. **(B–E)** Levels of MDA, T-SOD, GSH, and CAT in MLE-12 cells in different groups, n = 8. **(F)** DHE staining was used to detect ROS expression. Magnification: ×200, n = 3. **(G–I)** Levels of TNF-α, IL-1β, and IL-6 in the supernatant of MLE-12 cells in different groups, n = 8. **(J–T)** Protein levels of E-cadherin, N-cadherin, Vimentin, α-SMA, calpain-1, HSP90, TGF-β1, p-Smad2, p-Smad3, Smad2, and Smad3 were detected by Western blotting in MLE-12 cells, n = 3. The data are expressed as the means ± SEM. ^***^ *P* < 0.001 vs. the Con group, ^#^ *P* < 0.05, ^##^ *P* < 0.01 vs. the BLM group, ^$^ *P* < 0.05, ^$$^ *P* < 0.01 vs. the BLM + AS-IV group.

## 4 Discussion

In the present study, we demonstrate that Astragaloside IV significantly attenuated extracellular matrix accumulation, EMT, oxidative stress, and inflammatory responses in both *in vivo* and *in vitro* fibrotic models. Notably, using calpain-1 knockout mice, we established that calpain-1 serves as a critical upstream regulator of HSP90-mediated TGF-β1/Smad2/3 activation in pulmonary fibrosis. Furthermore, molecular docking, molecular dynamics simulations, and SPR experiments confirmed the direct binding interaction between Astragaloside IV and calpain-1, providing mechanistic insights into how this natural compound modulates the calpain-1/HSP90/TGF-β1 axis. These findings not only expand the known pharmacological profile of Astragaloside IV but also identify calpain-1 as a therapeutic target for pulmonary fibrosis intervention.

Inflammation and oxidative stress play crucial roles in the onset and progression of IPF (Wilson et al., 2010). Meanwhile, abnormal EMT is a critical determinant of fibrosis formation and a fundamental process in IPF progression (Yan et al., 2018). Following lung injury, damaged epithelial cells undergo continuous repair and healing, with inflammation playing a key role in IPF progression (Parker and Prince, 2011; Williams, 2003; Kinoshita and Goto, 2019). Injury to epithelial cells triggers the release of substantial inflammatory mediators and pro-fibrotic cytokines (Crosby and Waters, 2010). It has been shown that in patients with IPF, alveolar inflammatory cells produce large quantities of ROS, further contributing to the release of inflammatory mediators exacerbated by alveolar epithelial cell damage, thus advancing IPF progression (Cantin et al., 1987). The lung’s oxygen-rich environment facilitates BLM-induced ROS production, which perpetuates epithelial cell damage and leads to irreversible scar tissue formation and EMT (Bueno et al., 2020; Goll et al., 2003). Our research found that treatment with Astragaloside IV markedly reduced the production of TNF-α, IL-1β, IL-6, and CXCL1 in both animal models and cell cultures. Additionally, *in vivo* and *in vitro*, this treatment reduced MDA and ROS levels, increased SOD, GSH, and CAT levels, thereby enhancing antioxidant defense. During IPF progression, continuous damage to the lung epithelium causes excessive activation and aggregation of lung fibroblasts, leading to scar tissue formation and fibrotic lesions in the lungs (Pei et al., 2022). We cultured MLE-12 cells alongside MLF cells and observed that Astragaloside IV not only suppressed MLF cell growth and promoted apoptosis but also prevented the production of α-SMA and collagen I proteins and reduced extracellular matrix accumulation. Simultaneously, Astragaloside IV protected MLE-12 cells from damage. Furthermore, treatment with Astragaloside IV increased E-cadherin protein levels while decreasing the levels of N-cadherin, vimentin, fibronectin, collagen I, and α-SMA proteins. This resulted in a reduction in both EMT and fibrotic development. Notably, the therapeutic impact of Astragaloside IV increased with higher dosages. Astragaloside IV treatment also improved body weight, reduced lung coefficient and HYP content, and diminished collagen deposition in the lung tissue of mice. Our results indicated that Astragaloside IV significantly reduces inflammation, oxidative stress, and EMT induced by BLM.

HSP90 ensures the proper conformation of various proteins, including key mediators of signaling and transcriptional regulation, and plays a significant role in a wide array of pathophysiological processes (Sibinska et al., 2017). Studies have shown that HSP90 inhibitors attenuate oxidative stress in pulmonary atherosclerosis (Madrigal-Matute et al., 2012). In patients with IPF, HSP90 protein expression is elevated, and inflammation may promote HSP90 activation. Moreover, inhibition of HSP90 blocked the activation of the TGF-β1 signaling pathway (Sontake et al., 2017). TGF-β1 signals by activating the recruitment of downstream mediators Smad2 and Smad3, making it a strong pro-fibrotic mediator that promotes EMT (Cao et al., 2021; Marinova et al., 2020). Our results showed that BLM increased the protein expression of HSP90, TGF-β1, p-Smad2, and p-Smad3. Astragaloside IV has been shown to reduce the damage to MLE-12 cells treated with BLM, lower the levels of TNF-α, IL-1β, and IL-6, enhance the cells’ antioxidant defenses, and alleviate EMT in these cells. The therapeutic efficacy of Astragaloside IV was comparable to that of the inhibitors 17-AAG and SB525334. The findings indicated that Astragaloside IV mitigates oxidative stress, inflammation, and EMT induced by BLM, possibly through modulation of the HSP90/TGF-β1 signaling pathway.

The increased activity of calpain-1 has been observed in a range of pulmonary conditions (Liu et al., 2024; Deng et al., 2022). Our laboratory has demonstrated that Astragaloside IV can improve pulmonary arterial hypertension, brain injury, and endothelial dysfunction induced by chronic intermittent hypoxia through calpain-1-mediated inflammatory responses (Zhao et al., 2022; Sun et al., 2021; Meng et al., 2022). Furthermore, the binding of calpain-1 to HSP90 promotes the degradation of MMP2, exacerbating fibrosis formation caused by bladder cancer. Our results revealed that BLM upregulated the expression of calpain-1, whereas Astragaloside IV inhibited it. To determine whether Astragaloside IV ameliorates inflammation, oxidative stress, and EMT in BLM-induced pulmonary fibrosis via the calpain-1/HSP90/TGF-β1 signaling pathway, we utilized calpain-1 knockout mice and lentiviral overexpression of calpain-1 to transfect MLE-12 cells. The results showed that calpain-1 knockdown ameliorated fibrosis, attenuated inflammatory responses, increased antioxidant capacity, inhibited EMT, and decreased the expression of HSP90, TGF-β1, p-Smad2, and p-Smad3 in lung tissue of BLM-treated mice. Additionally, the effects of Astragaloside IV on MLE-12 cells were similar to those of MDL-28170, 17-AAG, and SB525334. After calpain-1 overexpression, Astragaloside IV lost its inhibitory effects on TNF-α, IL-1β, IL-6, MDA, ROS, antioxidant markers (SOD, GSH, CAT), and EMT. These results indicated that Astragaloside IV ameliorates BLM-induced pulmonary fibrosis through the calpain-1/HSP90/TGF-β1 signaling pathway. However, the present study did not directly assess the physical interaction between calpain-1 and HSP90 using co-immunoprecipitation or proximity ligation assays. Accordingly, our results should be interpreted as evidence that Astragaloside IV inhibits the calpain-1-associated HSP90/TGF-β1 signaling pathway, rather than definitive proof that it disrupts the physical interaction between calpain-1 and HSP90. Further studies are warranted to determine whether Astragaloside IV affects the formation or stability of the calpain-1/HSP90 complex. It is worth noting that the SPR analysis revealed a dissociation constant (KD) of 433 µM for the Astragaloside IV/calpain-1 interaction, indicating a relatively weak binding affinity. While this affinity is lower than that of typical high-potency synthetic inhibitors, it is not uncommon for natural products to exert therapeutic effects through moderate-affinity interactions, often relying on higher dosages or cumulative effects *in vivo*. In our study, the concentration of Astragaloside IV used in cell experiments (40 µM) and the high dosage administered in animal models (up to 100 mg/kg/day) likely facilitate sufficient target engagement to elicit biological responses. In addition, studies have demonstrated that Astragaloside IV inhibits intracellular Ca^2+^ in multiple models (Wang et al., 2025; Guo et al., 2016; Zhang et al., 2026). Since calpain-1 is a Ca^2+^-dependent proteolytic enzyme, Astragaloside IV may also indirectly regulate calpain-1. Furthermore, this weak binding does not preclude functional significance. Our data showing that calpain-1 overexpression abolishes the protective effects of Astragaloside IV strongly suggests that this interaction, potentially transient or allosteric in nature, is functionally critical for disrupting the calpain-1/HSP90/TGF-β1 axis.

In summary, the present study provides compelling evidence that targeted inhibition of the calpain-1/HSP90/TGF-β1 signaling axis significantly mitigates key pathological hallmarks of pulmonary fibrosis, including inflammation, oxidative stress, and EMT. We demonstrate for the first time that Astragaloside IV exerts its antifibrotic effects through calpain-1 inhibition, thereby disrupting HSP90-mediated TGF-β1/Smad2/3 activation (Figure 10). Mechanistically, further investigation is still required in the present study, given the absence of an *in vivo* CAPN1-KO + BLM + AS-IV group and *in vitro* combination groups of AS-IV with MDL-28170, 17-AAG, or SB525334. In addition, several limitations warrant consideration, including the need for long-term toxicity evaluation and validation in additional fibrosis models. Nevertheless, our results support Astragaloside IV as a promising natural compound for pulmonary fibrosis management and provide a scientific basis for its development as a dietary or nutraceutical intervention. Future studies should focus on optimizing Astragaloside IV bioavailability, exploring structure-activity relationships, and conducting clinical trials to translate these findings into therapeutic applications for patients with idiopathic pulmonary fibrosis.

**FIGURE 10:**
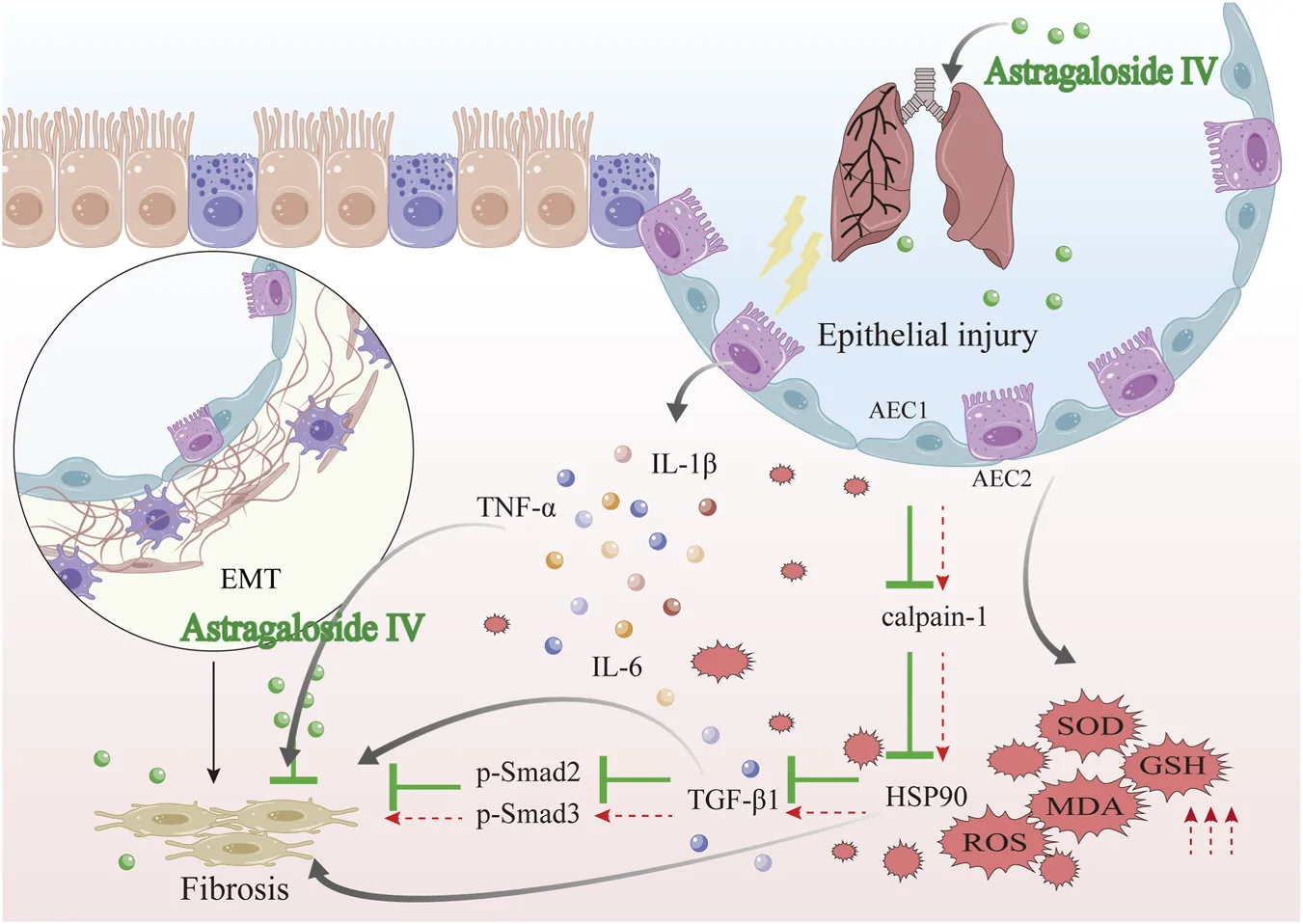
Astragaloside IV ameliorates bleomycin-induced pulmonary fibrosis by blocking the calpain-1/HSP90/TGF-β1 axis.

## Data Availability

The original contributions presented in the study are included in the article/Supplementary Material, further inquiries can be directed to the corresponding authors.

## References

[B1] Averna M. De Tullio R. Pedrazzi M. Bavestrello M. Pellegrini M. Salamino F. (2015). Interaction between calpain-1 and HSP90: new insights into the regulation of localization and activity of the protease. PLoS One 10 (1), e0116738. 10.1371/journal.pone.0116738 25575026 PMC4289065

[B2] Bueno M. Calyeca J. Rojas M. Mora A. L. (2020). Mitochondria dysfunction and metabolic reprogramming as drivers of idiopathic pulmonary fibrosis. Redox Biol. 33, 101509. 10.1016/j.redox.2020.101509 32234292 PMC7251240

[B3] Cantin A. M. North S. L. Fells G. A. Hubbard R. C. Crystal R. G. (1987). Oxidant-mediated epithelial cell injury in idiopathic pulmonary fibrosis. J. Clin. Invest. 79 (6), 1665–1673. 10.1172/JCI113005 3034979 PMC424497

[B4] Cao J. Xu T. Zhou C. Wang S. Jiang B. Wu K. (2021). NR4A1 knockdown confers hepatoprotection against ischaemia-reperfusion injury by suppressing TGFbeta1 *via* inhibition of CYR61/NF-kappaB in mouse hepatocytes. J. Cell. Mol. Med. 25 (11), 5099–5112. 10.1111/jcmm.16493 33942481 PMC8178266

[B5] Chatterjee R. Chatterjee J. (2020). ROS and oncogenesis with special reference to EMT and stemness. Eur. J. Cell Biol. 99 (2-3), 151073. 10.1016/j.ejcb.2020.151073 32201025

[B6] Crosby L. M. Waters C. M. (2010). Epithelial repair mechanisms in the lung. Am. J. Physiol.-Lung Cell. Mol. Physiol. 298 (6), L715–L731. 10.1152/ajplung.00361.2009 20363851 PMC2886606

[B7] Da Silva T. U. Pougy K. D. C. Albuquerque M. G. Da Silva Lima C. H. Machado S. D. P. (2022). Development of parameters compatible with the CHARMM36 force field for [Fe(4)S(4)](2+) clusters and molecular dynamics simulations of adenosine-5'-phosphosulfate reductase in GROMACS 2019. J. Biomol. Struct. Dyn. 40 (8), 3481–3491. 10.1080/07391102.2020.1847687 33183173

[B8] Deng H. Tian X. Sun H. Liu H. Lu M. Wang H. (2022). Calpain-1 mediates vascular remodelling and fibrosis *via* HIF-1alpha in hypoxia-induced pulmonary hypertension. J. Cell. Mol. Med. 26 (10), 2819–2830. 10.1111/jcmm.17295 35365973 PMC9097838

[B9] Goll D. E. Thompson V. F. Li H. Wei W. Cong J. (2003). The calpain system. Physiol. Rev. 83 (3), 731–801. 10.1152/physrev.00029.2002 12843408

[B10] Guan Y. Zhang J. Cai X. Cai Y. Song Z. Huang Y. (2024). Astragaloside IV inhibits epithelial-mesenchymal transition and pulmonary fibrosis *via* lncRNA-ATB/miR-200c/ZEB1 signaling pathway. Gene 897, 148040. 10.1016/j.gene.2023.148040 38065426

[B11] Guo H. Cao A. Chu S. Wang Y. Zang Y. Mao X. (2016). Astragaloside IV attenuates podocyte apoptosis mediated by endoplasmic reticulum stress through upregulating sarco/endoplasmic reticulum Ca(2+)-ATPase 2 expression in diabetic nephropathy. Front. Pharmacol. 7, 500. 10.3389/fphar.2016.00500 28066247 PMC5174081

[B12] Huang D. Shi S. Wang Y. Wang X. Shen Z. Wang M. (2022). Astragaloside IV alleviates PM2.5-caused lung toxicity by inhibiting inflammasome-mediated pyroptosis *via* NLRP3/caspase-1 axis inhibition in mice. Biomed. Pharmacother. 150, 112978. 10.1016/j.biopha.2022.112978 35462332

[B13] Jiang L. Zhang J. Monticone R. E. Telljohann R. Wu J. Wang M. (2012). Calpain-1 regulation of matrix metalloproteinase 2 activity in vascular smooth muscle cells facilitates age-associated aortic wall calcification and fibrosis. Hypertension 60 (5), 1192–1199. 10.1161/HYPERTENSIONAHA.112.196840 23006733 PMC3487400

[B14] Karabacak Atay C. Dilek O. Tilki T. Dede B. (2023). A novel imidazole-based azo molecule: synthesis, characterization, quantum chemical calculations, molecular docking, molecular dynamics simulations and ADMET properties. J. Mol. Model. 29 (8), 226. 10.1007/s00894-023-05625-1 37405575

[B15] Kinoshita T. Goto T. (2019). Molecular mechanisms of pulmonary fibrogenesis and its progression to lung cancer: a review. Int. J. Mol. Sci. 20 (6). 10.3390/ijms20061461 30909462 PMC6471841

[B16] Koli K. Myllarniemi M. Keski-Oja J. Kinnula V. L. (2008). Transforming growth factor-beta activation in the lung: focus on fibrosis and reactive oxygen species. Antioxid. Redox Signal. 10 (2), 333–342. 10.1089/ars.2007.1914 17961070

[B17] Li X. Peng J. Sun Z. Tian H. Duan X. Liu L. (2016). Chinese medicine CGA formula ameliorates DMN-Induced liver fibrosis in rats *via* inhibiting MMP2/9, TIMP1/2 and the TGF-beta/Smad signaling pathways. Acta Pharmacol. Sin. 37 (6), 783–793. 10.1038/aps.2016.35 27133300 PMC4954767

[B18] Li L. Jin R. Ji L. (2024). Pachymic acid ameliorates bleomycin-induced pulmonary fibrosis through inhibiting endoplasmic reticulum stress in rats. Environ. Toxicol. 39 (12), 5382–5390. 10.1002/tox.23824 37163307

[B19] Liu Y. Tang B. Lu M. Wang H. (2023). Astragaloside IV improves pulmonary arterial hypertension by increasing the expression of CCN1 and activating the ERK1/2 pathway. J. Cell. Mol. Med. 27 (5), 622–633. 10.1111/jcmm.17681 36762748 PMC9983322

[B20] Liu Y. Tang B. Wang H. Lu M. (2024). Otud6b induces pulmonary arterial hypertension by mediating the Calpain-1/HIF-1alpha signaling pathway. Cell. Mol. Life Sci. 81 (1), 258. 10.1007/s00018-024-05291-3 38878112 PMC11335297

[B21] Liu S. Xi Q. Li X. Liu H. (2025). Mitochondrial dysfunction and alveolar type II epithelial cell senescence: the destroyer and rescuer of idiopathic pulmonary fibrosis. Front. Cell. Dev. Biol. 13, 1535601. 10.3389/fcell.2025.1535601 40230412 PMC11994736

[B22] Lu J. Wang Q. Zhou Y. Lu X. Liu Y. Wu Y. (2017). AstragalosideIV against cardiac fibrosis by inhibiting TRPM7 channel. Phytomedicine 30, 10–17. 10.1016/j.phymed.2017.04.002 28545665

[B23] Madrigal-Matute J. Fernandez-Garcia C. E. Gomez-Guerrero C. Lopez-Franco O. Munoz-Garcia B. Egido J. (2012). HSP90 inhibition by 17-DMAG attenuates oxidative stress in experimental atherosclerosis. Cardiovasc. Res. 95 (1), 116–123. 10.1093/cvr/cvs158 22547655

[B24] Man V. H. Nguyen P. H. Derreumaux P. (2017). High-resolution structures of the amyloid-beta 1-42 dimers from the comparison of four atomistic force fields. J. Phys. Chem. B 121 (24), 5977–5987. 10.1021/acs.jpcb.7b04689 28538095 PMC5944325

[B25] Marinova M. Solopov P. Dimitropoulou C. Colunga Biancatelli R. M. L. Catravas J. D. (2020). Post-treatment with a heat shock protein 90 inhibitor prevents chronic lung injury and pulmonary fibrosis, following acute exposure of mice to HCl. Exp. Lung Res. 46 (6), 203–216. 10.1080/01902148.2020.1764148 32400213

[B26] Meng Y. Yu S. Zhao F. Liu Y. Wang Y. Fan S. (2022). Astragaloside IV alleviates brain injury induced by hypoxia *via* the Calpain-1 signaling pathway. Neural Plast. 2022, 6509981. 10.1155/2022/6509981 36510594 PMC9741538

[B27] Ono Y. Saido T. C. Sorimachi H. (2016). Calpain research for drug discovery: challenges and potential. Nat. Rev. Drug Discov. 15 (12), 854–876. 10.1038/nrd.2016.212 27833121

[B28] Parker D. Prince A. (2011). Innate immunity in the respiratory epithelium. Am. J. Respir. Cell. Mol. Biol. 45 (2), 189–201. 10.1165/rcmb.2011-0011RT 21330463 PMC3175551

[B29] Pei X. Zheng F. Li Y. Lin Z. Han X. Feng Y. (2022). Niclosamide ethanolamine salt alleviates idiopathic pulmonary fibrosis by modulating the PI3K-mTORC1 pathway. Cells 11 (3), 346. 10.3390/cells11030346 35159160 PMC8834116

[B30] Ren J. Vaid T. M. Lee H. Ojeda I. Johnson M. E. (2023). Evaluation of interactions between the hepatitis C virus NS3/4A and sulfonamidobenzamide based molecules using molecular docking, molecular dynamics simulations and binding free energy calculations. J. Comput. Aided. Mol. Des. 37 (1), 53–65. 10.1007/s10822-022-00490-1 36427108 PMC9839505

[B31] Sadatpour O. Kavosi H. Mahmoudi M. Mohammadi M. Saffar H. Farhadi E. (2026). Molecular mechanisms of pulmonary fibrosis: the interaction of epithelial-mesenchymal transition and AMPK pathways in a bleomycin-induced model. Iran. J. Allergy Asthma Immunol. 25 (1), 80–92. 10.18502/ijaai.v25i1.20441 41674175

[B32] Shapovalov I. Harper D. Greer P. A. (2022). Calpain as a therapeutic target in cancer. Expert Opin. Ther. Targets. 26 (3), 217–231. 10.1080/14728222.2022.2047178 35225722

[B33] Sibinska Z. Tian X. Korfei M. Kojonazarov B. Kolb J. S. Klepetko W. (2017). Amplified canonical transforming growth factor-beta signalling *via* heat shock protein 90 in pulmonary fibrosis. Eur. Respir. J. 49 (2). 10.1183/13993003.01941-2015 28182573

[B34] Sontake V. Wang Y. Kasam R. K. Sinner D. Reddy G. B. Naren A. P. (2017). Hsp90 regulation of fibroblast activation in pulmonary fibrosis. JCI Insight 2 (4), e91454. 10.1172/jci.insight.91454 28239659 PMC5313067

[B35] Storr S. J. Carragher N. O. Frame M. C. Parr T. Martin S. G. (2011). The calpain system and cancer. Nat. Rev. Cancer. 11 (5), 364–374. 10.1038/nrc3050 21508973

[B36] Suleiman M. R. Wang H. Huang D. Wang H. Joseph J. Huang T. (2021). Discovery of small molecule inhibitors through pharmacophore modeling, molecular docking, molecular dynamics simulation and experimental validation against myeloid cell leukemia-1 (Mcl-1). J. Biomol. Struct. Dyn. 39 (7), 2512–2525. 10.1080/07391102.2020.1749132 32228162

[B37] Sun Y. Lu M. Sun T. Wang H. (2021). Astragaloside IV attenuates inflammatory response mediated by NLRP-3/calpain-1 is involved in the development of pulmonary hypertension. J. Cell. Mol. Med. 25 (1), 586–590. 10.1111/jcmm.15671 33295020 PMC7810938

[B38] Tan W. Tan Q. Wang T. Lian M. Zhang L. Cheng Z. (2017). Calpain 1 regulates TGF-beta1-induced epithelial-mesenchymal transition in human lung epithelial cells *via* PI3K/Akt signaling pathway. Am. J. Transl. Res. 9 (3), 1402–1409. 28386365 PMC5376030

[B39] Thannickal V. J. Fanburg B. L. (2000). Reactive oxygen species in cell signaling. Am. J. Physiol.-Lung Cell. Mol. Physiol. 279 (6), L1005–L1028. 10.1152/ajplung.2000.279.6.L1005 11076791

[B40] Tomcik M. Zerr P. Pitkowski J. Palumbo-Zerr K. Avouac J. Distler O. (2014). Heat shock protein 90 (Hsp90) inhibition targets canonical TGF-Beta signalling to prevent fibrosis. Ann. Rheum. Dis. 73 (6), 1215–1222. 10.1136/annrheumdis-2012-203095 23661493

[B41] Wang X. Wang Y. Huang D. Shi S. Pei C. Wu Y. (2022). Astragaloside IV regulates the ferroptosis signaling pathway *via* the Nrf2/SLC7A11/GPX4 axis to inhibit PM2.5-mediated lung injury in mice. Int. Immunopharmacol. 112, 109186. 10.1016/j.intimp.2022.109186 36115280

[B42] Wang X. Chen X. Wang Y. He X. Li L. Wang X. (2024). Astragaloside IV alleviates inflammation and improves myocardial metabolism in heart failure mice with preserved ejection fraction. Front. Pharmacol. 15, 1467132. 10.3389/fphar.2024.1467132 39640484 PMC11618538

[B43] Wang J. Shi L. Wang C. Yao L. Li G. Wang S. (2025). Astragaloside depresses compound action potential in sciatic nerve of frogs involved in L-type Ca(2+)-channel dependent mechanism. Nat. Prod. Res. 39 (17), 4947–4956. 10.1080/14786419.2024.2353388 38824425

[B44] Williams M. C. (2003). Alveolar type I cells: molecular phenotype and development. Annu. Rev. Physiol. 65, 669–695. 10.1146/annurev.physiol.65.092101.142446 12428023

[B45] Wilson M. S. Madala S. K. Ramalingam T. R. Gochuico B. R. Rosas I. O. Cheever A. W. (2010). Bleomycin and IL-1beta-mediated pulmonary fibrosis is IL-17A dependent. J. Exp. Med. 207 (3), 535–552. 10.1084/jem.20092121 20176803 PMC2839145

[B46] Wu M. Li K. Wu J. Zhang Q. Ma X. Dai W. (2026). Astragaloside IV: a multipotent phytochemical for treating fibrotic diseases. Int. J. Mol. Med. 57 (2), 1–33. 10.3892/ijmm.2025.5721 PMC12768476 41456499

[B47] Xiao H. Cao Y. Wang Z. Liu C. (2026). Recent advances in astragaloside IV modulate immune response and skin cells promoting wound healing. Tissue Eng. Part B Rev., 19373341251381830. 10.1177/19373341251381830 41051944

[B48] Yan L. Song F. Li H. Li Y. Li J. He Q. (2018). Submicron emulsion of cinnamaldehyde ameliorates bleomycin-induced idiopathic pulmonary fibrosis *via* inhibition of inflammation, oxidative stress and epithelial-mesenchymal transition. Biomed. Pharmacother. 102, 765–771. 10.1016/j.biopha.2018.03.145 29604596

[B49] Yu W. Sun L. Yang H. (2016). Inhibitory effects of astragaloside IV on bleomycin-induced pulmonary fibrosis in rats *via* attenuation of oxidative stress and inflammation. Inflammation 39 (5), 1835–1841. 10.1007/s10753-016-0420-5 27525425

[B50] Zhang M. Wang W. Liu K. Jia C. Hou Y. Bai G. (2023). Astragaloside IV protects against lung injury and pulmonary fibrosis in COPD by targeting GTP-GDP domain of RAS and downregulating the RAS/RAF/FoxO signaling pathway. Phytomedicine 120, 155066. 10.1016/j.phymed.2023.155066 37690229

[B51] Zhang J. Lu M. Li C. Yan B. Xu F. Wang H. (2024). Astragaloside IV mitigates hypoxia-induced cardiac hypertrophy through calpain-1-mediated mTOR activation. Phytomedicine 125, 155250. 10.1016/j.phymed.2023.155250 38295664

[B52] Zhang Q. Zhao N. Wei S. Lu M. Chen K. (2026). Astragaloside IV prevents calpain-1-mediated cardiac hypertrophy and fibrosis induced by diabetes. Front. Cardiovasc. Med. 13, 1670472. 10.3389/fcvm.2026.1670472 41725932 PMC12916589

[B53] Zhao F. Meng Y. Wang Y. Fan S. Liu Y. Zhang X. (2022). Protective effect of astragaloside IV on chronic intermittent hypoxia-induced vascular endothelial dysfunction through the calpain-1/SIRT1/AMPK signaling pathway. Front. Pharmacol. 13, 920977. 10.3389/fphar.2022.920977 35983375 PMC9381017

[B54] Zhu M. Zhao L. Zhang X. Zhao R. (2024). Astragaloside IV restrains pulmonary fibrosis progression *via* the circ_0008898/miR-211-5p/HMGB1 axis. Chem. Biol. Drug Des. 103 (3), e14508. 10.1111/cbdd.14508 38514749

[B55] Zoz D. F. Lawson W. E. Blackwell T. S. (2011). Idiopathic pulmonary fibrosis: a disorder of epithelial cell dysfunction. Am. J. Med. Sci. 341 (6), 435–438. 10.1097/MAJ.0b013e31821a9d8e 21613930 PMC3103044

