# Supplementary material for: Blockade of the calpain-1/HSP90/TGF-β1 axis by Astragaloside IV ameliorates bleomycin-induced pulmonary fibrosis
Source: Front Pharmacol. 2026 Oct 14;17:1864914. doi: 10.3389/fphar.2026.1864914 (PMC13713264; doi:10.3389/fphar.2026.1864914)
Supplement: Supplementary file 1 [file Supplementaryfile1.docx]

Supplementary Material

# 1 Supplementary Figures

**Supplementary Figure 1. Validation of CAPN1 overexpression efficiency in cells.** (A) Representative Western blot bands showing calpain‑1 protein levels in Con, pLV‑NC and pLV‑CAPN1 groups. (B) Quantitative analysis of relative calpain‑1 protein expression normalized to β‑actin. Con, blank control; pLV‑NC, cells infected with empty lentivirus; pLV‑CAPN1, cells infected with CAPN1‑overexpressing lentivirus. Data are presented as means ± SEM. ****** P < 0.01 versus pLV‑NC group.

**Supplementary Figure 2.** Astragaloside IV and CAPN1 knockdown ameliorated BLM‑induced alteration of SP‑c and Vimentin expression in lung tissues. (A) Quantitative analysis of fluorescence intensity for Vimentin (B)Quantitative analysis of fluorescence intensity for SP-c.The data are expressed as the means ± SEM. ***P < 0.001 vs. the Con group, ^##^P < 0.01 vs. the BLM group.

**Supplementary Figure 3.** Comparison of the therapeutic effects of Astragaloside and pirfenidone within the vivo. (A) Western blotting to detect N-cadherin, E-cadherin and α-SMA protein expression in lung tissues, n=3. (B-G) Expression of inflammatory factors in mouse lung tissue and BALF, n=8. (H-J) Detection of MDA, T-SOD, and GSH content in lung tissues of each group of mice, n=8. The data are expressed as the means ± SEMs. ****P* < 0.001 vs. the Con group, ^#^*P* < 0.05, ^##^*P* < 0.01 vs. the BLM group.

**Supplementary Figure 4.** Comparative Analysis of the Therapeutic Effects of Astragaloside IV and Pirfenidone in MLE-12 Cells. (A) Western blotting to detect N-cadherin, E-cadherin and α-SMA protein expression in MLE-12 cells, n=3. (B-D) Detection of MDA, T-SOD, and GSH content in MLE-12 cells, n=8. (E-G) Expression of inflammatory factors in MLE-12 cells, n=8. The data are expressed as the means ± SEMs. ****P* < 0.001 vs. the Con group, ^#^*P* < 0.05, ^##^*P* < 0.01 vs. the BLM group.
